# Diagnostic performance of deep learning-based automatic white matter hyperintensity segmentation for classification of the Fazekas scale and differentiation of subcortical vascular dementia

**DOI:** 10.1371/journal.pone.0274562

**Published:** 2022-09-15

**Authors:** Leehi Joo, Woo Hyun Shim, Chong Hyun Suh, Su Jin Lim, Hwon Heo, Woo Seok Kim, Eunpyeong Hong, Dongsoo Lee, Jinkyeong Sung, Jae-Sung Lim, Jae-Hong Lee, Sang Joon Kim

**Affiliations:** 1 Department of Radiology, Korea University Guro Hospital, Seoul, Republic of Korea; 2 Department of Radiology and Research Institute of Radiology, Asan Medical Center, University of Ulsan College of Medicine, Seoul, Republic of Korea; 3 Department of Convergence Medicine, Asan Medical Center, University of Ulsan College of Medicine, Ulsan, Republic of Korea; 4 VUNO Inc., Seoul, Republic of Korea; 5 Department of Neurology, Asan Medical Center, University of Ulsan College of Medicine, Seoul, Republic of Korea; IRCCS San Raffaele Scientific Research Institute, ITALY

## Abstract

**Purpose:**

To validate the diagnostic performance of commercially available, deep learning-based automatic white matter hyperintensity (WMH) segmentation algorithm for classifying the grades of the Fazekas scale and differentiating subcortical vascular dementia.

**Methods:**

This retrospective, observational, single-institution study investigated the diagnostic performance of a deep learning-based automatic WMH volume segmentation to classify the grades of the Fazekas scale and differentiate subcortical vascular dementia. The VUNO Med-DeepBrain was used for the WMH segmentation system. The system for segmentation of WMH was designed with convolutional neural networks, in which the input image was comprised of a pre-processed axial FLAIR image, and the output was a segmented WMH mask and its volume. Patients presented with memory complaint between March 2017 and June 2018 were included and were split into training (March 2017–March 2018, n = 596) and internal validation test set (April 2018–June 2018, n = 204).

**Results:**

Optimal cut-off values to categorize WMH volume as normal vs. mild/moderate/severe, normal/mild vs. moderate/severe, and normal/mild/moderate vs. severe were 3.4 mL, 9.6 mL, and 17.1 mL, respectively, and the AUC were 0.921, 0.956 and 0.960, respectively. When differentiating normal/mild vs. moderate/severe using WMH volume in the test set, sensitivity, specificity, and accuracy were 96.4%, 89.9%, and 91.7%, respectively. For distinguishing subcortical vascular dementia from others using WMH volume, sensitivity, specificity, and accuracy were 83.3%, 84.3%, and 84.3%, respectively.

**Conclusion:**

Deep learning-based automatic WMH segmentation may be an accurate and promising method for classifying the grades of the Fazekas scale and differentiating subcortical vascular dementia.

## Introduction

White matter hyperintensity (WMH) is known to represent accumulated tissue damage of white matter and is detected on T2-weighted images and Fluid Attenuated Inversion Recovery (FLAIR) images showing hyperintense signal intensities [1, 2]. It is associated with executive dysfunction regardless of its location [3], and a potential association exists between WMH and diverse diseases, including cerebrovascular, as well as other neurological or psychiatric diseases [4–7]. Cognitive impairment is also associated with WMH [8, 9].

The volume of WMH is thought to represent the size of cerebral small vessel disease burden, and a larger volume of WMH is reported to have an association with poor functional outcome or treatment response in patients with acute ischemic stroke [10–13]. For volume estimation, visual assessment has been mainly performed such as the Fazekas scale [14] in clinical practice, which is also easy and quick to perform. However, reliable assessment is difficult and sufficient experience is needed owing to the inherent heterogeneous nature in size, number, shape, and location of WMH, which increases with age and cannot be objective [15]. These problems remain in manual segmentation of WMH, which is time-consuming and labor-intensive.

In recent years, automated methods for measuring the volume of WMH were tried using various methods of deep learning [16, 17], and convolutional neural networks (CNN) have become one of the main methods with variable performance [18–22]. Park et al. [22] used a U-Net model adopting a multi-scale approach that obtains significant features from intermediate decoder layers to segment WMH. Wu et al. [21] developed a skip connection U-Net that adds skip connections to standard U-Net and achieved faster convergence speed and improved segmentation performance. The VUNO Med-DeepBrain is one of the commercially available segmentation models, which employed a 2D U-Net using only T2-FLAIR MRI for development and focused on treating highly unbalanced WMH labels, mainly owing to deep WMH by applying generalized dice loss [23]. The difficulty of deep WMH underestimation was also suggested in another article [24] that utilized domain adaptation methods to develop a robust segmentation model regardless of scanner or sequence types.

Meanwhile, subcortical vascular dementia (or subcortical ischemic vascular dementia) is a major form of vascular dementia that results from complete or incomplete infarction of cerebral subcortical structures due to small artery disease [25]. The main clinical features include cognitive impairment [26], which is related to disruption of the prefrontal-subcortical circuit by the accumulation of lacunar infarctions and white matter ischemia [27, 28] and interruption of cholinergic pathways traversing the subcortical white matter [1, 29]. Cholinergic pathway comprise neurons regulating synthesis and release of acetylcholine, which is a ubiquitous neurotransmitter having a central role in neurotransmission and cognition [30]. One of the diagnostic criteria is severe WMH on T2-weighted and FLAIR images [31].

In this study, we aimed to evaluate the diagnostic performance of a deep learning-based automatic WMH segmentation algorithm for classifying grades of the Fazekas scale and differentiating subcortical vascular dementia, which has not been evaluated yet.

## Materials and methods

### Patient inclusion

All data were fully anonymized before access, and the institutional review boards (IRB) approved this observational study of a single tertiary referral hospital (IRB No. 2020–1352). The requirement for informed consent was waived given the retrospective design. A computerized search of electronic medical records was performed to identify consecutive patients attending the outpatient neurologic memory clinic to evaluate memory impairment between March 2017 and June 2018. To validate a segmentation model, the dataset was split into the training set (March 2017–March 2018, n = 596) and internal validation test set (April 2018–June 2018, n = 204). The eligibility criteria included patients who: (a) presented with memory complaints, (b) underwent brain MRI as part of their initial evaluation, (c) had available electronic medical records, (d) did not have a previous history of the cerebral infarct, and (e) did not have other white matter abnormality such as metabolic encephalopathy or postoperative change. Brain MRI was performed within 1 month of presentation.

### Brain MRI protocol

Brain MRI was performed on 3.0 T units (Ingenia, Philips Medical Systems, Best, the Netherlands) using a 32-channel sensitivity-encoding head coil. High-resolution anatomical three-dimensional (3D) volume images were acquired using a 3D gradient-echo T1-weighted sequence in the sagittal plane. The detailed parameters were as follows: repetition time (TR), 9.6 ms; an echo time (TE), 4.6 ms; a flip angle, 8°; a field of view (FOV), 224 mm × 224 mm; slice thickness, 1 mm with no gap; and a matrix size, 224 × 224. Two-dimensional (2D) axial FLAIR images were obtained as follows: TR, 9000 ms; TE, 125 ms; FOV, 220 mm × 220 mm; slice thickness, 4 mm with no gap; and a matrix size, 256 × 256.

### MRI analysis and reference standards

The FLAIR images were reviewed in consensus by two neuroradiologists with 10 and 31 years of neuroradiology experience, respectively. They were blinded to all the clinical information. WMH was categorized as normal, mild, moderate, or severe according to the Fazekas scale in both the periventricular and deep white matter [3]. Periventricular WMH was graded as follows: (a) normal, absence; (b) mild, "caps" or pencil-thin lining; (c) moderate, smooth "halo"; and (d) severe, irregular periventricular hyperintensity extending into the deep white matter [3]. Deep WMH was graded as follows: (a) normal, absence; (b) mild, punctate foci; (c) moderate, beginning confluence of foci; and (d) severe, large confluent areas [3]. In case of discordance, a consensus was made by two neuroradiologists for each periventricular WMH and deep WMH. In addition, the representative grade of WMH for each case was determined as maximal grading of either periventricular or deep white matter. Sensitivity was defined as the proportion of a test positive (mild/moderate/severe, moderate/severe or severe from DeepBrain), conditioned on a true positive (mild/moderate/severe, moderate/severe or severe, based on a consensus by two neuroradiologists). Interobserver agreement between the two neuroradiologists was determined for all patients.

The patients who fulfilled the following criteria were diagnosed with subcortical vascular dementia by clinicians [31]: (a) met the criteria for Vascular dementia by the Diagnostic and Statistical Manual of Mental Disorders—fourth edition [32]; (b) had at least one focal neurologic sign on the Focal Neurologic Sign Score evaluation [31]; (c) had severe WMH on T2-weighted and FLAIR images, defined as periventricular WMH with a cap or rim >10 mm in maximum diameter, deep WMH with extensive or diffusely confluent form ≥25 mm in maximum diameter [31]. Patients with images that showed territorial, or watershed infarctions were excluded. All relevant data are within the manuscript and its Supporting Information files (S1 Appendix).

### Deep learning-based WMH segmentation algorithm

The VUNO Med-DeepBrain (version 1.0.1, VUNO Inc., Seoul, South Korea), which has been commercially available in South Korea since June 2019 and Europe since June 2020, was used for the deep learning-based segmentation system. Deep learning algorithms can provide the segmentation of detailed regions of the T1-weighted brain MRI and WMH regions of the FLAIR image. The software extracts the segmented 104 brain regions masked image whose space, direction, orientation information equal to the original 3D T1 MR Image. The total white matter volume is calculated using space information and the number of the pixel of the total segmented white matter region.

**Fig 1:**
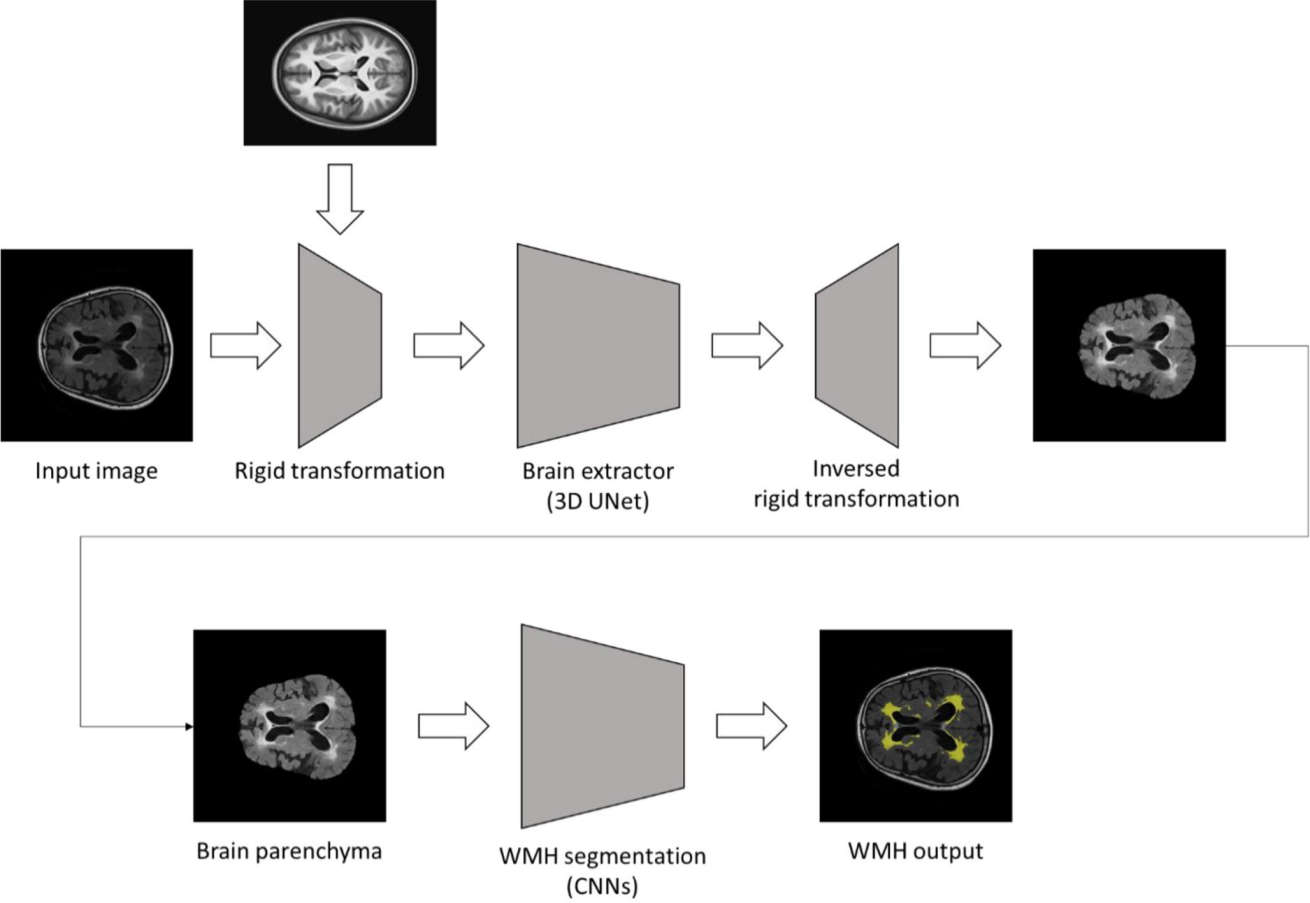
A schematic diagram of the deep learning-based WMH segmentation algorithm. The deep learning-based WMH segmentation algorithm is consisting of two independent processes. First, brain extraction is conducted with two rigid transformation and in-house brain extraction algorithm using 3D U-Net. Second, in-house convolutional neural networks segment WMH from the preprocessed brain parenchyma image.

**Fig 2:**
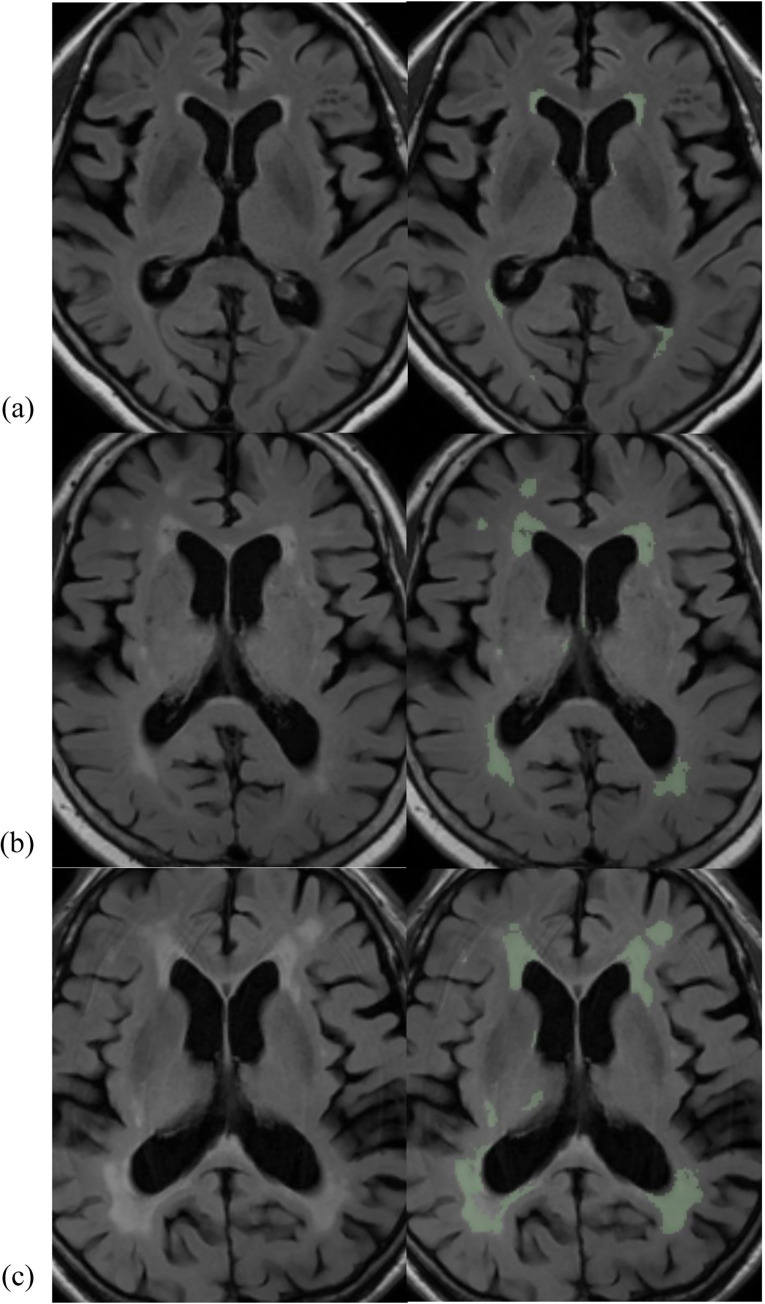
Three pairs of a pre-processed FLAIR image and a mask of segmented WMH with total WMH volume of each case in different Fazekas categories (a-c) (WMH volume: a, 4.11 mL; b, 20.59 mL; c, 47.69 mL, the ratio of WMH volume / total white matter volume: a, 1.09%; b, 5.18%; c, 9.94%). The last pair is the images of a subcortical vascular dementia patient (c). FLAIR, fluid-attenuated inversion recovery.

The system for segmentation of WMH was designed with CNN, in which the input image was comprised of a pre-processed FLAIR image, and the output was a segmented WMH mask and its volume (Fig 1). Brain parenchyma were extracted from the FLAIR images using the in-house brain extraction tool with a rigid transformation for template matching and a 3D UNet deep learning model. The UNet architecture is an end-to-end CNN that conducts high-performance biomedical image segmentation [33]. The UNet architecture contains two modules: encoder and decoder. The encoder modules are a classic stack of CNN and max pooling layers that analyze the context that represents the implicit features of the input image. Whereas the decoder modules are a symmetric stack of transposed CNN that perform localization. The UNet applies a skip connection between the encoder modules’ feature and decoder modules’ feature for improved localization performance. UNet is widely used, primary in medical image segmentation, because of its high performance. The 3D UNet architecture consists of 4 encoder blocks and 3 decoder blocks using 3D convolution blocks (3D-conv block), 3D up convolution blocks (3D-upconv block) and a 1x1 3D convolution layer. The 3D-conv block sequentially includes 3D convolution– 3D batch normalization–Rectified Linear Unit (ReLU) activation– 3D convolution– 3D batch normalization–ReLU activation. First 3D-conv block has padding 3 and kernel size 5 for the convolution layers, otherwise has padding 1 and kernel size 3. For the encoder, we perform 3D-conv block calculation and then conduct a 3D max-pooling layer (kernel size 2, stride 2) to re-duce an image dimension. For the decoder, we act 3D-upconv block (3D convolution Transpose– 3D batch normalization–ReLU activation) and then perform the 3D-conv block. Finally, the 1x1 3D convolution layer is applied to produce an output mask. This model has [8, 16, 32, 64] channels for the encoder blocks [8, 16, 32], channels for the de-coder blocks, and a final layer has a one channel. A WMH segmentation model has a similar architecture to the 3D UNet, however, there are some differences. This model is based on 2D layers (2D convolution, 2D convolution Transpose, 2D batch normalization, 2D max-pooling layers) and consists of 5 encoder blocks and 4 decoder blocks. It has [16, 32, 64, 128, 256] channels for the encoder blocks [128, 64, 16, 32], channels for the decoder blocks. The WMH segmentation model was developed for effective segmentation for large masks such as periventricular WMH and small masks such as deep WMH [33]. The pre-processed image passed through the CNN to generate a segmentation mask. The overall processing time is 2 minutes per case. Fig 2 shows three examples of a pre-processed FLAIR image and a mask of segmented WMH, with total WMH volume of each case classified according to Fazekas. This deep learning-based segmentation system was trained using ADAM optimizer with learning rate 1e-4, 32 batch size, 100 epochs, generalized dice loss function. Elastic deformation, mirror transformation was used for the image augmentation technique. The data regarding the segmentation model (DeepBrain), which is presented in this study, are owned by VUNO Inc.

### Statistical analyses

The primary outcome was the diagnostic performance of automatic WMH volume and volume ratio for classifying the grades of Fazekas scale with its optimal cut-off values. WMH volume ratio was defined as automatic WMH volume/total white matter volume × 100. One-way analysis of variance was done among groups with different Fazekas scales in both training and test sets. The interobserver agreement of the Fazekas scale for each periventricular WMH and deep WMH between two neuroradiologists was calculated using weighted Kappa. Optimal cut-off values for determining the Fazekas scale were obtained from receiver operating characteristic (ROC) curves, with the sensitivity, specificity, and area under each curve (AUC) calculated using the Youden index [34], defined as sensitivity + specificity– 1 (values ranged from -1 to +1). The secondary outcome was the diagnostic performance of automatic WMH volume and volume ratio for differentiating subcortical vascular dementia with its optimal cut-off values. All statistical analyses were performed using MedCalc version 18.6 (MedCalc Software, Ostend, Belgium), with *P* values < 0.05 defined as statistically significant. Meanwhile, we obtained a Dice similarity coefficient score using our test set by manual segmentation to evaluate the competence of the model.

## Results

### Patient demographics

**Fig 3:**
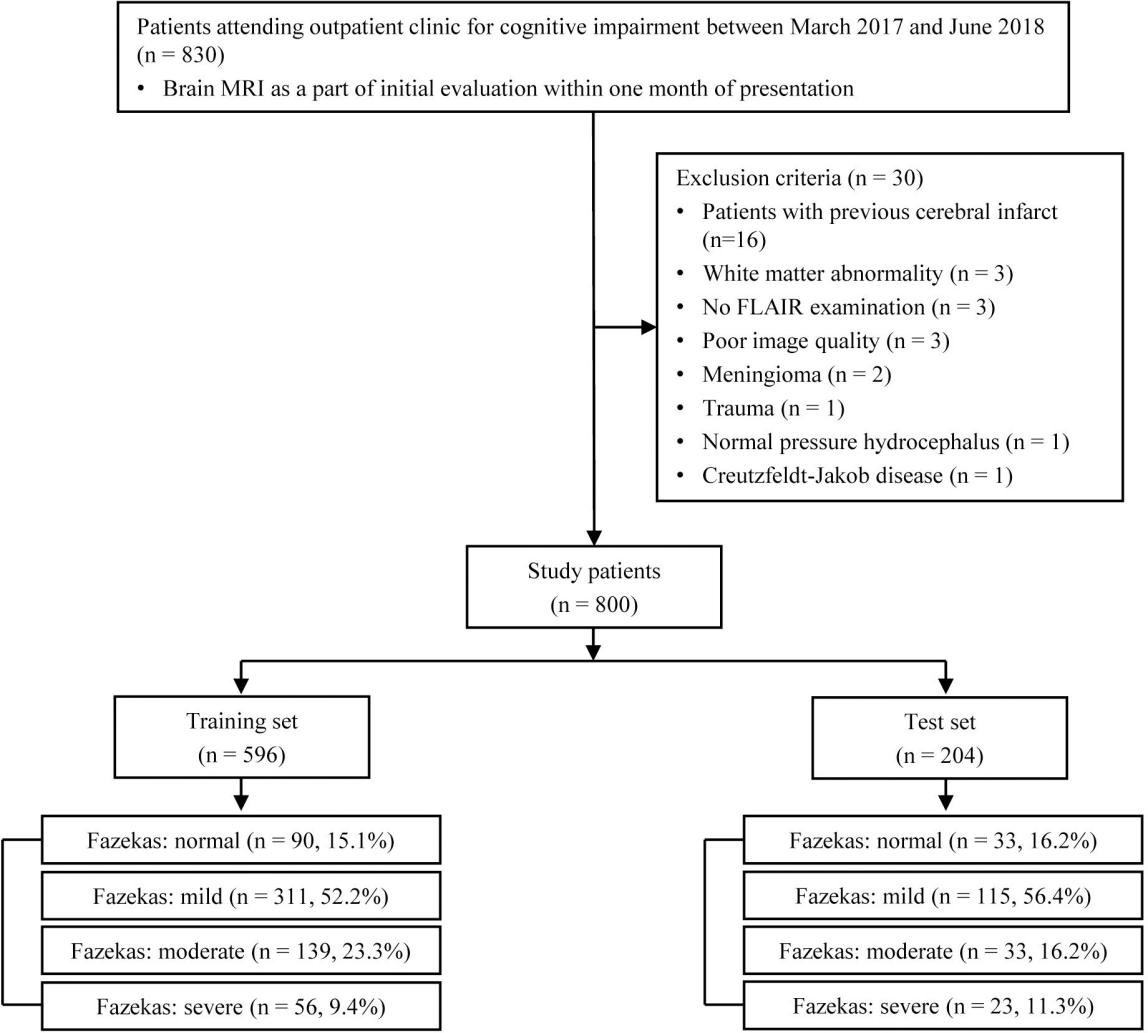
Flow diagram showing the selection process of patients and their Fazekas scale. FLAIR, fluid-attenuated inversion recovery; MRI, magnetic resonance imaging.

Between March 2017 and June 2018, 830 patients attended the outpatient clinic for memory complaints. Patients with previous cerebral infarct (n = 16), white matter abnormality (n = 3), no FLAIR examination (n = 3), poor image quality (n = 3), meningioma (n = 2), trauma (n = 1), normal pressure hydrocephalus (n = 1), and Creutzfeldt-Jakob disease (n = 1) were excluded, leaving the remaining 800 consecutive patients who were included in this analysis (Fig 3). The mean age ± standard deviation (SD) of the training and test sets was 69.1 years ± 10.4 and 69.4 years ± 10.8, respectively; 350 and 129 patients were women in the training and test sets, respectively. The demographic characteristics of patients in training and test set based on the Fazekas scale are shown in Table 1. In the training set (n = 596), there were 90, 311, 139, and 56 patients categorized as normal, mild, moderate, and severe. Moderate and severe categories accounted for 32.7% (195 of 596). In the test set (n = 204), there were 33, 115, 33, and 23 patients categorized as normal, mild, moderate, and severe, respectively. Moderate and severe categories constituted 27.5% (56 of 204).

**Table 1:** Characteristics of patients based on the Fazekas scale.

		Fazekas scale				
*Training set*	All patients (n = 596)	Normal (n = 90)	Mild (n = 311)	Moderate (n = 139)	Severe (n = 56)	*P* values
Age (year)	69.1 ± 10.4	57.2 ± 11.8	68.3 ± 8.9	75.1 ± 6.6	76.0 ± 6.8	< 0.001
No. of male patients	246	55	118	46	27	
No. of female patients	350	35	193	93	29	
Education (year)	10.2 ± 5.1	12.7 ± 4.5	10.3 ± 4.9	9.0 ± 5.3	8.8 ± 5.4	< 0.001
MMSE score	24.7 ± 5.2	27.3 ± 3.4	25.3 ± 5.0	23.3 ± 5.1	21.4 ± 6.0	< 0.001
CDR	0.6 ± 0.4	0.4 ± 0.3	0.5 ± 0.4	0.7 ± 0.4	0.7 ± 0.4	< 0.001
WMH volume (mL)	11.7 ± 13.3	2.2 ± 1.7	7.0 ± 9.3	18.3 ± 9.9	36.5 ± 13.1	< 0.001
WMH volume/total white matter volume ×100 (%)	2.9 ± 3.4	0.5 ± 0.4	1.7 ± 2.4	4.6 ± 2.5	9.2 ± 3.7	< 0.001
*Test set*	All patients (n = 204)	Normal (n = 33)	Mild (n = 115)	Moderate (n = 33)	Severe (n = 23)	*P* values
Age (year)	69.4 ± 10.8	57.0 ± 12.8	70.0 ± 8.7	76.2 ± 6.2	74.7 ± 8.0	< 0.001
No. of male patients	75	9	46	14	6	
No. of female patients	129	24	69	19	17	
Education (year)	10.0 ± 4.9	11.8 ± 5.0	10.7 ± 4.8	7.7 ± 4.0	8.5 ± 5.1	0.002
MMSE score	25.3 ± 5.1	28.7 ± 2.6	25.4 ± 4.9	24.3 ± 4.8	21.4 ± 6.2	< 0.001
CDR	0.4 ± 0.4	0.1 ± 0.2	0.4 ± 0.4	0.5 ± 0.5	0.7 ± 0.4	< 0.001
WMH volume (mL)	10.7 ± 13.6	1.8 ± 1.2	5.5 ± 3.3	17.7 ± 6.3	39.3 ± 20.5	< 0.001
WMH volume/total white matter volume ×100 (%)	2.7 ± 3.4	0.4 ± 0.3	1.3 ± 0.8	4.5 ± 1.8	9.9 ± 5.1	< 0.001

Note—Unless otherwise specified, data are mean data ± standard deviation.

MMSE, Mini-Mental State Examination; CDR, Clinical Dementia Rating; WMH, white matter hyperintensity

Among the 596 patients in the training set, 28 (4.7%) were diagnosed with subcortical vascular dementia. The mean age ± SD was 76.7 years ± 6.3, and 15 patients were women. The mean duration of education in years ± SD was 9.4 ± 5.2, the mean MMSE score ± SD was 18.7 ± 5.3, and the mean Clinical Dementia Rating (CDR) was 0.9 ± 0.3. The mean total white matter volume ± SD was 392.0 mL ± 56.0. Among the 204 patients in the test set, 6 (2.9%) were diagnosed with subcortical vascular dementia. The mean age ± SD was 77.3 years ± 7.0, and 6 patients were women. The mean duration of education in years ± SD was 5.8 ± 3.0, the mean MMSE score ± SD was 18.2 ± 4.4, and the mean CDR was 1.0 ± 0.5. The mean total white matter volume ± SD was 380.0 mL ± 29.7.

### Optimal cut-off values for classifying Fazekas scale

**Fig 4:**
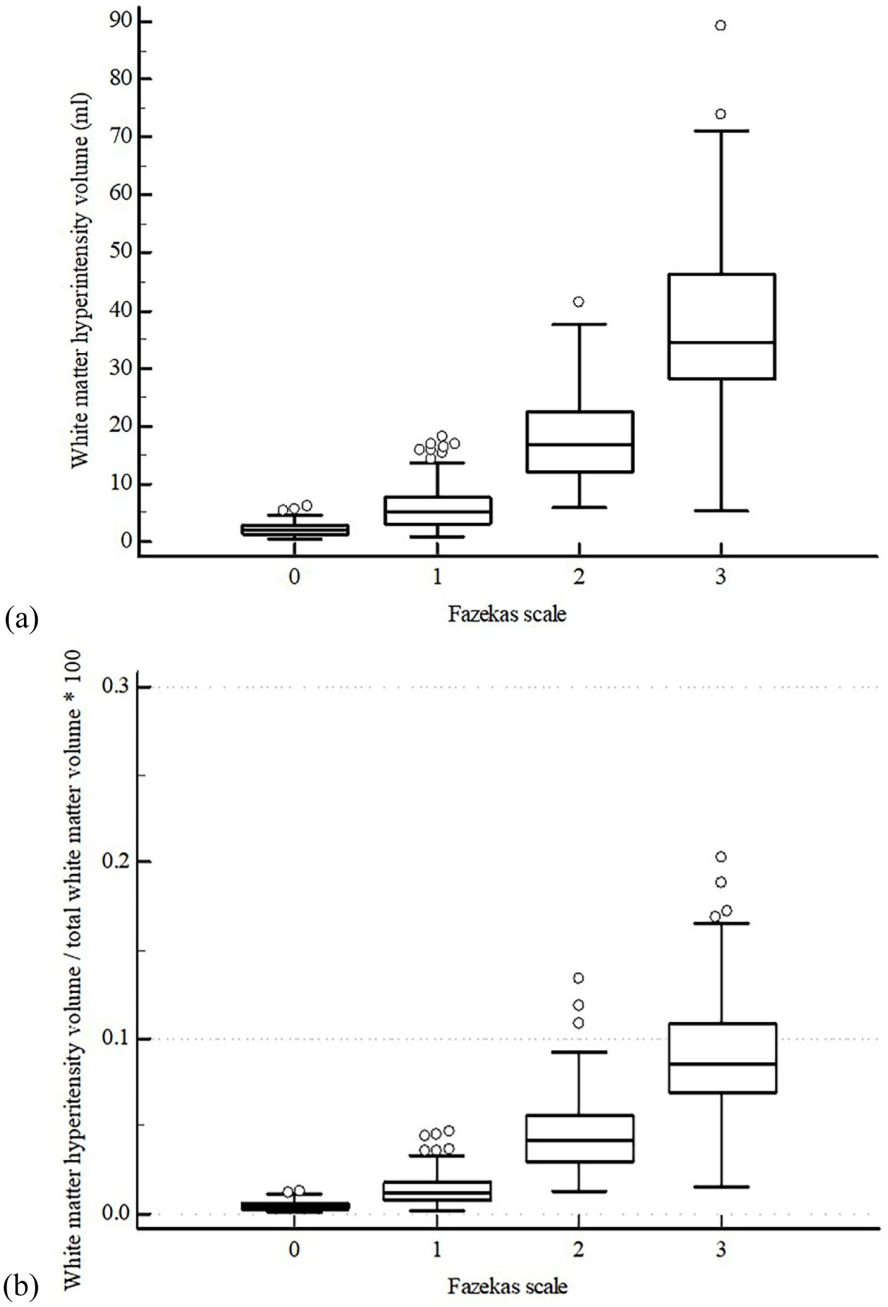
WMH volume (a) and WMH volume ratio (b) for Fazekas categories.

The optimal cut-off values for classifying the grades of Fazekas scale using WMH volume and volume ratio are described in Table 2. The mean WMH volume of the four previously described categories were 2.3 mL, 6.6 mL, 18.2 mL, and 37.3 mL, respectively (Fig 4). In the training set (n = 596), optimal cut-off values of WMH volume for differentiating normal vs. mild/moderate/severe, normal/mild vs. moderate/severe, and normal/mild/moderate vs. severe were 3.4 mL, 9.6 mL and 17.1 mL, respectively, and AUCs were 0.921 (95% CI: 0.896–0.941), 0.956 (95% CI: 0.936–0.971), and 0.960 (95% CI: 0.941–0.975), respectively.

**Table 2:** Diagnostic performance of WMH volume for differentiating Fazekas scale with its cut-off values.

Fazekas scale	Cut-off	Sensitivity (95% CI)	Specificity (95% CI)	Accuracy (95% CI)
WMH volume (mL)				
Normal vs. mild/moderate/severe	3.4 mL	79.5% (72.7%–85.3%)	90.9% (75.7%–98.1%)	81.4% (75.3%–86.5%)
Normal/mild vs. moderate/severe	9.6 mL	96.4% (87.7%–99.6%)	89.9% (83.8%–94.2%)	91.7% (87.0%–95.1%)
Normal/mild/moderate vs. severe	17.1 mL	87.0% (66.4%–97.2%)	90.6% (85.4%–94.4%)	90.2% (85.3%–93.9%)
WMH volume/total white matter volume ×100 (%)				
Normal vs. mild/moderate/severe	0.7%	83.6% (77.2%–88.8%)	84.9% (68.1%–94.9%)	83.8% (78.0%–88.6%)
Normal/mild vs. moderate/severe	2.5%	92.9% (82.7%–98.0%)	92.6% (87.1%–96.2%)	92.6% (88.2%–95.8%)
Normal/mild/moderate vs. severe	4.6%	87.0% (66.4%–97.2%)	91.7% (86.7%–95.3%)	91.2% (86.4%–94.7%)

CI, confidence interval; WMH, white matter hyperintensity

The mean WMH volume ratio of the four categories were 0.5%, 1.6%, 4.6%, and 9.4%, respectively (Fig 4). In the training set (n = 596), optimal cut-off values of WMH volume ratio for differentiating normal vs. mild/moderate/severe, normal/mild vs. moderate/severe, and normal/mild/moderate vs. severe were 0.7%, 2.5%, and 4.6%, respectively, and AUCs were 0.924 (95% CI: 0.900–0.944), 0.960 (95% CI: 0.941–0.975), and 0.960 (95% CI: 0.941–0.974), respectively. ROC curves are available in the supplementary section (S1–S6 Figs).

In terms of interobserver agreement between the two neuroradiologists for classifying Fazekas scale of periventricular and deep WMH, the weighted Kappa values were 0.815 (95% CI: 0.785–0.846) and 0.852 (95% CI: 0.825–0.879), respectively.

### Diagnostic performance for classifying Fazekas scale

The diagnostic performance of WMH volume and volume ratio for classifying the grades of the Fazekas scale with the cut-off values are described in Table 2. When differentiating normal/mild vs. moderate/severe using WMH volume in the test set (n = 204), sensitivity, specificity, and accuracy were 96.4% (54/56, 95% CI: 87.7%–99.6%), 89.9% (133/148, 95% CI: 83.8%–94.2%), and 91.7% (187/204, 95% CI: 87.0%–95.1%), respectively. When differentiating normal/mild/moderate vs. severe using WMH volume in the test set (n = 204), sensitivity, specificity, and accuracy were 87.0% (20/23, 95% CI: 66.4%–97.2%), 90.6% (164/181, 95% CI: 85.4%–94.4%), and 90.2% (184/204, 95% CI: 85.3%–93.9%), respectively.

When differentiating normal/mild vs. moderate/severe using WMH volume ratio in the test set (n = 204), sensitivity, specificity, and accuracy were 92.9% (52/56, 95% CI: 82.7%–98.0%), 92.6% (137/148, 95% CI: 87.1%–96.2%), and 92.6% (189/204, 95% CI: 88.2%–95.8%), respectively. When differentiating normal/mild/moderate vs. severe using WMH volume ratio in the test set (n = 204), sensitivity, specificity, and accuracy were 87.0% (20/23, 95% CI: 66.4%–97.2%), 91.7% (166/181, 95% CI: 86.7%–95.3%), and 91.2% (186/204, 95% CI: 86.4%–94.7%), respectively.

### Optimal cut-off values for differentiating subcortical vascular dementia

Among 596 patients of training set, 4.7% (28/596) has diagnosed as subcortical vascular dementia with (n = 13) or without combined Alzheimer’s disease (n = 15). For distinguishing subcortical vascular dementia from others using WMH volume, optimal cut-off value was 17.0 mL and AUC was 0.931 (95% CI: 0.907–0.950). For distinguishing subcortical vascular dementia from others using WMH volume ratio, optimal cut-off value was 3.4 and AUC was 0.934 (95% CI: 0.911–0.953).

### Diagnostic performance for differentiating subcortical vascular dementia

Among the 204 patients of the test set, 2.9% (6/204) were diagnosed as subcortical vascular dementia with (n = 4) or without combined Alzheimer’s disease (n = 2). For distinguishing subcortical vascular dementia from others using WMH volume, sensitivity, specificity, and accuracy were 83.3% (5/6, 95% CI: 35.9%–99.6%), 84.3% (167/198, 95% CI: 78.5%–89.1%), and 84.3% (172/204, 95% CI: 78.6%–89.0%), respectively. For distinguishing subcortical vascular dementia from others using WMH volume ratio, sensitivity, specificity, and accuracy were 100.0% (6/6, 95% CI: 54.1%–100.0%), 79.8% (158/198, 95% CI: 73.5%–85.2%), and 80.4% (164/204, 95% CI: 74.3%–85.6%), respectively.

## Discussion

The current study investigated the diagnostic performance of a deep learning-based automatic WMH volume segmentation to classify the grades of the Fazekas scale and differentiate subcortical vascular dementia. In this study, optimal cut-off values for determining WMH volume as normal vs. mild/moderate/severe, normal/mild vs. moderate/severe and normal/mild/moderate vs. severe were 3.4 mL, 9.6 mL and 17.1 mL, and AUCs were 0.921 (95% CI: 0.896–0.941), 0.956 (95% CI: 0.936–0.971), and 0.960 (95% CI: 0.941–0.975). The WMH volume ratio showed similar results. When differentiating normal/mild vs. moderate/severe using WMH volume in the test set, sensitivity, specificity, and accuracy were 96.4%, 89.9%, and 91.7%, respectively. For differentiating subcortical vascular dementia using WMH volume and volume ratio, optimal cut-off values were 17.0 mL and 3.4 with AUCs being 0.931 (95% CI: 0.907–0.950) and 0.934 (95% CI: 0.911–0.953), respectively. In the test set for evaluating diagnostic performance of differentiating subcortical vascular dementia using WMH volume, sensitivity was 83.3% (5/6, 95% CI: 35.9%–99.6%), 84.3% (167/198, 95% CI: 78.5%–89.1%), and accuracy was 84.3% (172/204, 95% CI: 78.6%–89.0%). Therefore, the deep learning-based automatic WMH segmentation algorithm may be an accurate and promising method for classifying the grades of the Fazekas scale and differentiating subcortical vascular dementia.

The advantage of using the Fazekas scale is the easy perception of WMH burden because it is the most prevalent method for grading WMH lesions. However, it is a qualitative method and limited to accurately evaluating WMH volume using only 4 grades. Numerous attempts have been done for quantitative measurement of WMH using deep learning [16]. There was a MICCAI WMH segmentation challenge [35] in which the winner used a 2D U-Net model that applied an ensemble method and achieved a 0.80 dice similarity coefficient. DeepBrain, which also employed a 2D U-Net, focused on treating highly unbalanced WMH labels by applying generalized dice loss, and our model used only T2-FLAIR MRI for developing a WMH segmentation model. DeepBrain showed relatively high performance by achieving a 0.746 dice similarity coefficient score (median value) for our test set. However, direct comparison with the winner of the challenge model was not possible because the external test set of the competition has not been released.

We speculated that if suitable volume cut-off values can be evaluated with acceptable diagnostic performance based on a well-developed automatic volumetry, an automated and reliable Fazekas scale can be determined. We expect this would help the better perception of WMH burden in both quantitative and qualitative ways; moreover, it would facilitate follow-up of the burden of WMH. One study investigated the diagnostic performance of the automatic WMH segmentation method in distinguishing high Fazekas grades (score = 2 or 3) and low Fazekas grades (score = 0 or 1) using the Lesion Segmentation Tool software [36]. The AUC value from that study was 0.93, whereas our results showed that AUC was 0.981 (95% CI: 0.951–0.995), with the cut-off being 9.6 mL for differentiating normal/mild vs. moderate/severe. However, our study provided additional diagnostic performance with optimal cut-off values for classifying normal and severe Fazekas grades. We also evaluated the WMH volume ratio. Moreover, our study noted the diagnostic performance of WMH in differentiating subcortical vascular dementia from other subtypes of dementia, which had not been evaluated previously.

Subcortical vascular dementia is a type of vascular dementia which is the second most common cause of dementia following Alzheimer’s disease (AD) [37]. Two-thirds of patients with subcortical vascular dementia have pathologic features of AD, and one-third of patients with AD have vascular pathology, which implies that subcortical vascular dementia and AD may overlap pathologically [38–41]. However, the existence of pure subcortical vascular dementia was discovered based on the amyloid imaging, which represents significant extent of subcortical white matter ischemic changes without evidence of amyloid plaque deposition in the brain [42]. Apart from mixed type with AD, pure subcortical vascular dementia demonstrates less difficulties in verbal/visual memory-related tasks but more problem in executive functions [25, 43–46]. However, the diagnosis of subcortical vascular dementia was not made on the basis of the amyloid imaging in our study because it is not routinely performed in clinical practice. Rather, clinical and radiologic criteria were used by clinicians as previously described [31].

Our study has several limitations. First, because it was a retrospective in design, absence of bias in patient selection could not be guaranteed. Second, the study was performed in a single center. However, it was conducted with a large cohort of 800 consecutive patients. Further multi-centered prospective studies will be required. Third, the cut-off values may depend on the scanner and the algorithms used for the automatic WMH. Fourth, our study MRI protocol used 2D FLAIR images with 4 mm section thickness, thus, there might be a variation for obtaining volume measurement. Further study using 3D FLAIR images might be needed. Fifth, we could not evaluate the competence of the already developed and commercially available segmentation model. However, Dice similarity coefficient score was 0.714 ± 0.149 (mean ± SD) with the median of 0.746 using our test set.

## Conclusions

In conclusion, the deep learning-based automatic WMH segmentation algorithm may be an accurate and promising method for classifying the grades of the Fazekas scale and differentiating subcortical vascular dementia.

## Supporting information

S1 Appendix Relevant data. (PDF)

S1 Fig ROC curve for normal vs. mild/moderate/severe using an optimal cut-off of WMH. (TIF)

S2 Fig ROC curve for normal/mild vs. moderate/severe using an optimal cut-off of WMH. (TIF)

S3 Fig ROC for normal/mild/moderate vs. severe using an optimal cut-off of WMH. (TIF)

S4 Fig ROC for normal vs. mild/moderate/severe using an optimal cut-off of WMH volume ratio. (TIF)

S5 Fig ROC for normal/mild vs. moderate/severe using an optimal cut-off of WMH volume ratio. (TIF)

S6 Fig ROC for normal/mild/moderate vs. severe using an optimal cut-off of WMH volume ratio. (TIF)
