# Supplementary material for: Diagnostic performance of deep learning-based automatic white matter hyperintensity segmentation for classification of the Fazekas scale and differentiation of subcortical vascular dementia
Source: PLoS One. 2022 Sep 15;17(9):e0274562. doi: 10.1371/journal.pone.0274562 (PMC9477348; doi:10.1371/journal.pone.0274562)
Supplement: S1 Appendix — (PDF) [file pone.0274562.s001.pdf]

Age	Sex	Eduction	MMSE	CDR	WMH volume	(WMH volume)/ (WM volume)	FAZEKAS_MAX
64	M	16	26	0.5	8.5189	0.0188	1
50	F	16	17	1	5.4282	0.0157	3
57	M	12	4	3	6.7095	0.0151	1
72	F	12	28	0.5	6.4769	0.0147	1
72	F	2	20	1	4.1099	0.0109	1
80	F	0.5	19	0.5	30.1466	0.0946	3
74	F	9	27	0.5	68.2287	0.1657	3
71	M	16	30	0.5	6.6504	0.0150	1
80	F	8	25	0.5	32.7684	0.0882	3
68	F	11	25	0.5	5.0515	0.0115	1
91	F	9	28	0	2.8537	0.0077	1
75	M	16	26	0.5	5.9023	0.0126	1
45	F	12	25	0.5	3.1055	0.0085	1
86	F	3	18	1	13.8547	0.0395	2
72	F	14	28	0	31.3541	0.0919	2
76	M	9	30	0.5	8.5521	0.0219	1
75	M	16	30	0.5	5.7900	0.0134	1
68	F	9	29	0	6.7760	0.0177	1
70	F	12	28	0.5	3.2827	0.0074	1
69	F	12	22	0.5	5.0891	0.0140	1
66	F	9	19	0.5	3.5929	0.0088	1
73	F	6	28	0	6.3698	0.0153	1
74	F	6	28	0.5	2.4630	0.0058	1
71	F	6	26	0	16.9821	0.0340	2
86	M	16	26	0.5	10.1584	0.0228	1
76	M	12	24	0.5	19.8220	0.0473	2
63	F	7	28	0.5	2.4445	0.0051	1
74	F	1	22	0.5	26.5869	0.0662	3
68	F	13	30	0	4.3019	0.0090	1
74	F	6	26	0.5	5.9289	0.0155	1
75	M	16	27	0.5	5.7782	0.0115	1
83	F	7	24	1	13.2602	0.0361	2
74	F	6	18	1	26.0478	0.0605	3
64	M	12	28	0.5	8.2789	0.0184	1
56	M	16	29	0	2.1860	0.0044	1
75	M	18	30	0	10.5941	0.0240	1
74	F	2	18	0.5	4.1284	0.0115	1
84	M	5	4	2	35.8000	0.0820	3
56	M	12	30	0	7.4517	0.0145	1
74	F	6	29	0	7.9731	0.0200	2
76	F	6	19	0.5	13.9109	0.0374	2

69	F	16	24	1	2.5811	0.0064	1
61	M	16	28	0	3.2754	0.0065	1
30	M	16	30	0	1.2518	0.0024	0
61	F	12	30	0	1.0576	0.0023	1
72	F	6	30	0	11.6126	0.0274	2
72	M	12	25	0.5	14.3680	0.0332	1
83	M	16	29	0.5	9.7522	0.0211	1
79	F	6	15	1	10.3467	0.0293	1
75	F	7	28	0.5	28.2191	0.0732	3
62	M	12	28	0.5	2.3411	0.0047	1
63	F	6	26	0.5	14.3680	0.0306	2
63	F	12	30	0	1.9977	0.0044	0
56	F	18	27	0.5	2.6292	0.0056	1
81	F	4	14	0.5	4.5862	0.0130	1
85	F	0.5	13	1	11.2810	0.0306	1
79	F	4	19	0.5	1.6026	0.0046	1
66	F	12	29	0	3.8366	0.0088	1
85	M	6	22	0.5	10.1621	0.0280	2
49	M	16	30	0	2.3522	0.0045	1
88	F	0.5	19	0.5	5.6756	0.0169	1
71	M	16	30	0	5.5648	0.0117	1
66	M	2	23	1	21.7776	0.0569	2
76	M	1	28	0	22.6136	0.0644	2
54	F	16	29	0	1.0694	0.0027	0
57	F	16	25	0.5	1.7281	0.0040	0
78	F	9	26	0.5	7.4111	0.0159	1
71	F	0	17	0.5	1.9756	0.0044	0
81	F	12	26	0.5	15.6087	0.0427	2
88	F	6	18	1	20.9630	0.0679	2
70	M	12	25	0.5	7.3742	0.0165	1
73	M	16	30	0.5	4.1623	0.0089	1
62	M	12	28	0.5	2.7658	0.0058	1
76	M	9	26	0.5	10.6163	0.0247	2
74	F	5	17	1	14.7927	0.0352	2
66	F	4	17	1	15.7638	0.0413	3
77	M	9	23	1	89.1142	0.2310	3
68	F	6	30	0	10.7345	0.0274	2
75	F	12	27	0.5	3.6742	0.0104	1
65	M	18	25	0.5	16.8495	0.0308	1
70	M	6	29	0	9.8113	0.0228	2
79	F	9	24	0.5	3.2421	0.0083	1
50	F	14	27	0.5	2.1343	0.0048	0
69	M	6	26	0	6.0854	0.0113	0

64	F	16	22	0.5	5.6423	0.0132	1
64	F	12	28	0.5	3.2089	0.0075	0
77	F	8	29	0.5	11.6945	0.0252	2
68	M	12	16	1	4.7081	0.0124	1
79	F	12	23	0.5	11.6133	0.0298	1
70	F	2	28	0	12.8858	0.0289	2
71	F	6	26	0.5	3.1277	0.0067	1
76	F	15	26	0.5	49.1156	0.1197	3
87	M	16	25	0.5	26.3801	0.0660	2
67	F	16	30	0	0.6277	0.0017	0
84	F	6	17	0.5	73.9264	0.2031	3
70	F	12	25	0.5	7.2191	0.0180	1
77	F	12	28	1.5	8.3985	0.0209	1
62	F	14	19	0.5	4.9777	0.0118	1
73	F	6	20	0.5	9.9849	0.0301	1
87	F	6	12	2	21.5206	0.0616	3
64	F	9	29	0	5.6386	0.0133	1
82	F	0	9	1	5.3543	0.0157	1
79	F	6	28	0.5	7.5625	0.0216	1
74	M	16	25	0.5	7.3963	0.0171	1
74	M	16	29	0	4.4496	0.0105	1
81	F	6	14	1	4.9407	0.0147	1
69	M	14	24	0.5	45.2531	0.0936	3
80	F	6	25	0.5	28.1415	0.0701	3
75	F	14	30	0.5	1.8463	0.0052	1
56	F	9	26	0	2.5294	0.0058	1
77	M	16	28	0.5	46.2465	0.1212	3
73	M	16	29	0.5	7.2375	0.0157	1
73	M	13	15	0.5	71.1200	0.1497	3
69	F	6	26	0.5	5.4444	0.0147	1
66	F	6	27	0.5	13.9471	0.0372	3
56	F	16	19	1	2.8618	0.0072	1
66	F	6	29	0	5.3728	0.0136	1
79	F	0.5	16	0.5	32.4656	0.0844	3
64	F	16	26	0.5	1.3973	0.0040	1
76	M	7	28	0	13.0793	0.0277	1
66	M	12	28	0	4.0028	0.0098	1
85	F	0.5	19	0.5	8.5300	0.0237	1
80	F	9	15	2	32.0815	0.0818	2
70	M	12	23	0.5	5.3875	0.0142	1
77	M	16	29	0.5	13.2676	0.0322	1
60	M	19	30	0.5	2.7857	0.0056	1
70	M	6	25	0.5	4.8669	0.0109	1

69	M	18	28	0.5	39.3560	0.0957	3
63	M	17	29	0	5.4392	0.0100	1
60	M	14	27	0.5	3.3086	0.0063	1
76	M	6	25	0.5	4.7820	0.0118	1
57	M	13	26	0.5	3.2237	0.0068	0
76	M	12	29	0.5	13.5963	0.0342	2
83	F	6	21	0.5	4.6379	0.0136	1
83	F	3	26	0	3.2717	0.0095	0
69	F	6	26	0.5	3.0095	0.0075	1
75	F	8	30	0	3.8810	0.0088	1
65	M	16	28	0	2.3485	0.0054	1
78	F	12	21	1	35.2720	0.1008	3
72	F	16	30	0.5	3.9400	0.0109	1
83	M	16	22	0.5	30.0801	0.0828	2
78	F	6	29	0	26.5352	0.0616	2
67	M	9	24	0.5	19.7541	0.0524	2
68	M	6	29	0.5	5.4614	0.0105	1
50	F	14	28	0.5	2.3190	0.0053	1
80	F	5	12	2	17.6803	0.0447	2
51	F	9	17	1	5.8897	0.0139	1
72	F	14	23	0.5	4.1431	0.0112	1
78	M	12	27	0.5	3.7886	0.0103	0
81	M	4	19	1	18.8287	0.0523	2
79	M	12	27	0.5	17.7948	0.0476	2
63	M	6	30	0.5	2.9836	0.0067	1
68	F	10	29	0.5	1.6728	0.0045	1
69	M	16	26	0	5.1106	0.0106	1
73	M	6	28	0.5	7.0455	0.0147	1
78	M	16	26	0.5	18.0496	0.0470	1
66	F	9	29	0	1.4032	0.0033	1
58	F	9	27	0	2.0716	0.0049	1
76	M	7	22	0.5	3.8145	0.0090	1
63	F	12	30	0.5	4.1564	0.0094	1
78	M	12	28	0.5	3.6742	0.0074	1
69	F	6	25	0.5	9.6082	0.0240	1
80	F	6	25	0.5	46.2317	0.1201	3
76	M	16	29	0.5	11.7160	0.0263	1
67	F	6	27	0.5	3.0501	0.0069	1
77	F	13	25	0.5	2.4445	0.0056	1
75	M	6	23	0.5	22.9091	0.0522	2
72	F	8	30	0	1.8389	0.0044	1
71	M	14	28	0	16.8864	0.0400	2
67	F	3	28	0.5	2.6661	0.0066	1

62	M	16	29	0.5	5.5279	0.0125	1
84	F	10	27	0.5	6.4510	0.0159	1
45	F	12	28	0.5	0.7533	0.0016	0
62	F	16	30	0	4.3462	0.0110	1
59	F	9	27	0.5	6.0633	0.0144	1
76	F	1	20	0.5	3.2680	0.0094	1
60	F	14	23	0.5	7.1637	0.0171	1
67	F	10	23	0.5	9.7670	0.0226	1
65	F	12	29	0.5	42.2141	0.1015	3
66	F	16	30	0	4.5848	0.0096	0
78	F	5	20	0.5	18.2748	0.0498	2
67	F	13	28	0.5	2.3300	0.0060	0
68	M	16	28	0	4.4570	0.0104	1
61	F	6	27	0	2.0826	0.0051	1
74	F	12	29	0.5	17.4957	0.0432	2
75	F	15	19	0.5	3.8197	0.0081	1
87	M	0.5	20	0.5	11.9161	0.0256	1
72	F	6	28	0	5.2029	0.0153	1
92	M	14	21	1	37.8952	0.0926	3
63	M	14	30	0.5	4.3388	0.0094	1
64	M	14	29	0.5	3.5412	0.0081	1
73	M	6	20	0.5	3.7185	0.0100	1
73	F	3	20	1	25.0006	0.0638	3
77	M	20	29	0.5	8.7257	0.0181	1
65	F	9	28	0	0.6241	0.0017	0
67	M	12	28	0.5	3.0390	0.0072	1
77	F	0	20	0.5	1.8020	0.0048	1
58	F	9	17	1	4.5124	0.0121	1
74	F	12	27	0	1.5694	0.0040	1
63	M	16	29	0	5.3617	0.0101	1
60	M	9	19	1	3.4977	0.0088	1
70	F	6	19	0.5	10.2323	0.0293	2
56	F	6	23	0.5	1.5398	0.0039	1
56	M	16	28	0.5	2.7510	0.0064	1
84	M	11	26	0.5	42.3884	0.0863	3
67	F	12	25	0.5	6.2073	0.0155	1
73	F	14	28	0.5	46.4739	0.1219	3
61	F	12	28	0.5	8.4524	0.0180	1
69	M	18	27	0.5	3.4378	0.0077	1
51	M	6	21	0.5	2.6107	0.0047	0
76	M	3	23	0.5	10.1732	0.0253	2
61	F	6	23	0.5	4.7044	0.0119	1
75	M	12	30	0.5	6.5212	0.0152	1

81	M	16	25	1	63.3064	0.1626	3
66	F	2	27	0.5	2.0620	0.0054	1
80	F	16	30	0.5	33.0859	0.0834	3
78	M	14	26	0.5	2.4593	0.0059	1
66	F	6	27	0.5	4.3484	0.0113	1
74	F	2	18	0.5	53.8296	0.1685	3
75	F	12	27	0.5	9.2463	0.0255	1
79	F	6	19	1	34.0054	0.1085	2
56	F	12	22	0.5	2.2562	0.0054	0
76	F	12	24	0.5	8.3638	0.0217	1
76	F	6	26	0.5	6.7723	0.0180	1
71	M	16	30	0.5	5.3839	0.0123	1
84	F	0	15	1	26.7420	0.0819	2
81	M	16	26	0.5	8.6038	0.0192	1
69	F	6	30	0	2.6735	0.0065	0
59	F	9	30	0	2.8286	0.0066	1
64	F	9	26	0.5	14.7040	0.0295	2
48	M	14	29	0.5	2.9246	0.0058	0
56	M	12	28	0	1.8168	0.0037	1
78	M	16	30	0.5	6.2996	0.0126	1
88	M	18	28	0.5	40.8405	0.0855	3
77	F	9	27	0.5	9.7448	0.0303	2
79	F	12	30	0	10.1769	0.0229	1
53	F	16	28	0	1.2740	0.0031	0
85	F	3	24	0.5	3.6926	0.0105	1
67	M	7	24	0.5	3.6631	0.0089	1
74	M	14	26	0.5	6.2036	0.0137	1
75	F	12	27	0.5	6.2885	0.0156	1
82	F	6	27	1	24.2786	0.0709	2
75	M	18	29	0.5	5.3115	0.0127	1
84	F	7	22	0.5	3.0516	0.0089	1
65	F	12	30	0	10.8563	0.0212	1
54	M	14	30	0.5	3.9363	0.0080	1
70	F	6	30	0.5	3.8366	0.0081	1
67	F	0.5	27	0.5	6.1113	0.0134	1
59	F	6	23	0.5	3.2717	0.0084	1
55	F	12	22	0.5	7.6068	0.0172	1
77	M	16	20	1	10.2832	0.0237	1
84	M	12	20	0.5	15.8598	0.0374	2
73	M	12	26	0.5	6.4436	0.0139	1
78	M	7	26	0.5	10.7492	0.0262	1
65	F	16	29	0.5	2.7510	0.0067	1
62	M	9	25	0.5	9.3756	0.0195	1

54	M	12	19	1	63.1938	0.1233	1
54	F	12	25	0.5	4.4422	0.0114	1
62	F	6	25	0.5	4.6047	0.0100	1
70	F	6	5	3	5.1032	0.0107	1
55	M	14	29	0	4.0619	0.0081	1
84	M	16	15	1	42.6779	0.1131	3
80	M	12	26	0.5	9.9516	0.0288	2
87	F	12	26	0.5	8.3712	0.0234	2
78	M	16	28	0.5	5.7753	0.0160	1
69	M	9	26	0.5	7.8247	0.0187	1
60	F	12	26	0.5	2.8950	0.0080	1
61	F	5	30	0.5	7.0345	0.0178	2
65	M	7	23	1	23.1011	0.0528	2
61	F	6	3	2	3.9105	0.0108	1
56	F	12	10	2	10.2286	0.0274	1
73	M	12	24	1	15.9115	0.0362	1
82	F	0.5	19	1	22.1410	0.0584	2
63	M	6	29	0.5	3.9105	0.0082	1
66	M	6	24	0	0.7681	0.0026	0
85	M	12	27	0.5	21.5059	0.0560	2
71	M	7	25	0.5	17.9794	0.0453	2
72	F	12	28	0	5.9525	0.0158	1
70	F	9	23	0.5	11.8127	0.0327	2
74	F	9	22	0.5	10.4317	0.0279	2
64	M	16	29	0.5	2.8027	0.0056	0
65	F	12	29	0	12.0749	0.0261	2
79	F	5	30	0.5	5.1955	0.0127	1
42	M	12	26	0.5	1.7651	0.0032	0
76	M	6	21	1	66.6778	0.1881	3
79	F	6	24	0.5	23.5516	0.0574	2
66	F	6	26	0.5	1.3072	0.0029	0
82	F	14	27	0.5	11.0225	0.0402	2
51	F	16	30	0	0.9785	0.0021	0
74	M	16	21	1	4.4430	0.0112	1
63	F	16	28	0	2.0679	0.0057	1
65	F	12	29	0	8.5263	0.0174	1
67	F	12	26	0.5	3.8773	0.0091	1
83	F	12	23	0.5	14.8776	0.0358	2
81	F	3	15	1	22.7687	0.0584	3
61	F	2	22	0.5	1.4438	0.0035	0
73	F	18	26	0.5	17.2401	0.0486	2
52	M	16	30	0	1.2370	0.0029	0
60	F	12	29	0.5	3.1341	0.0068	1

74	F	6	23	0.5	6.9163	0.0169	1
44	F	14	29	0.5	1.7798	0.0033	0
64	M	16	30	0.5	1.0044	0.0023	1
79	M	14	22	0.5	30.8593	0.0734	3
76	M	11	24	0.5	7.4739	0.0184	1
63	M	17	30	0.5	2.5775	0.0054	1
65	M	6	23	0.5	0.9859	0.0020	0
67	F	6	16	2	16.7276	0.0388	2
74	F	2	18	0.5	5.5796	0.0149	1
70	M	12	28	0	1.8131	0.0041	0
42	F	12	22	1	3.0021	0.0069	1
83	F	10	21	2	8.3343	0.0263	2
70	F	3	14	1	5.5020	0.0135	1
75	F	1	23	0.5	9.5920	0.0233	2
69	F	12	24	0.5	2.4925	0.0065	1
77	M	6	28	0.5	15.4278	0.0382	2
74	M	16	28	0.5	2.4224	0.0051	1
45	F	12	28	0.5	1.0155	0.0023	0
75	F	7	20	0.5	69.0844	0.1712	1
64	F	12	29	0.5	0.7208	0.0019	0
60	F	16	29	0	1.8463	0.0043	0
69	F	16	28	0.5	4.9703	0.0125	1
77	F	0.5	23	0.5	51.3644	0.1372	3
73	F	9	20	1	30.7559	0.0770	3
74	M	13	23	1	8.3690	0.0190	1
52	F	14	28	0.5	2.0273	0.0040	1
76	M	12	24	0.5	36.5349	0.0834	3
76	F	7	24	0.5	10.4797	0.0280	2
76	M	12	23	0.5	27.0094	0.0680	2
78	M	16	30	0.5	10.6606	0.0241	1
67	F	6	27	0.5	8.9989	0.0179	1
78	F	3	21	0.5	5.8048	0.0164	1
45	M	14	30	0.5	1.0192	0.0024	0
81	F	12	29	0	13.8067	0.0366	2
85	F	5	26	1	54.8872	0.1496	3
60	F	12	18	0.5	3.4674	0.0080	1
60	F	6	27	0.5	1.7318	0.0045	0
85	F	9	12	2	67.4717	0.1723	3
82	F	0.5	17	0.5	26.0441	0.0728	2
69	F	2	27	0.5	6.5803	0.0144	1
62	F	6	29	0.5	3.2901	0.0073	1
67	F	3	18	2	4.2724	0.0106	1
79	M	0.5	20	1	15.3244	0.0301	1

69	F	6	30	0.5	2.5073	0.0060	1
35	F	14	30	0	0.4357	0.0011	0
62	M	9	29	0.5	7.9982	0.0195	1
77	M	18	23	0.5	12.8503	0.0356	1
76	M	3	21	0.5	17.3000	0.0457	2
40	F	14	30	0	1.1299	0.0028	0
64	F	6	27	0.5	1.3072	0.0033	1
84	F	16	22	0.5	7.6142	0.0220	2
86	M	8	25	1	37.6980	0.1044	3
65	F	12	29	0	2.1639	0.0050	0
49	M	12	29	0.5	1.2555	0.0025	0
56	F	12	29	0	2.3633	0.0056	1
79	M	12	28	0.5	25.6785	0.0644	2
72	F	6	26	0.5	14.6154	0.0372	2
59	M	12	30	0	2.4283	0.0050	1
60	M	12	13	1	4.5382	0.0117	1
62	M	5	21	0.5	8.6851	0.0221	1
76	F	9	27	0.5	8.4598	0.0246	1
64	F	10	26	0.5	2.9541	0.0076	1
81	M	14	28	0.5	7.9872	0.0178	1
79	F	12	24	0.5	4.1446	0.0116	1
74	F	2	19	0.5	27.9753	0.0595	2
65	M	12	29	0.5	4.0176	0.0085	0
41	F	18	30	0.5	2.4408	0.0054	0
64	F	6	5	1	12.6325	0.0366	1
76	F	10	20	1	53.0372	0.1591	3
63	F	6	28	0	8.7035	0.0213	1
62	M	18	29	0	4.0767	0.0083	1
80	M	12	18	1	20.4793	0.0567	2
79	M	9	17	0.5	46.7339	0.0958	3
63	F	12	24	0.5	2.9061	0.0064	0
61	M	9	24	0.5	69.5026	0.1361	1
62	F	4	30	0	1.2762	0.0031	1
57	F	12	30	0	3.1203	0.0077	1
77	M	12	24	2	30.5653	0.0655	2
81	F	9	22	1	10.4354	0.0295	1
76	F	12	27	0.5	6.0485	0.0167	1
58	F	16	30	0	3.0132	0.0063	1
72	F	6	25	1	69.0697	0.2216	1
79	F	14	25	0.5	15.7306	0.0450	1
74	M	21	19	1	62.9870	0.1342	2
74	F	12	27	0	3.7185	0.0099	1
62	M	15	23	0.5	4.4053	0.0092	1

73	F	3	25	0.5	11.5358	0.0273	2
78	F	12	28	1	8.3158	0.0249	2
61	F	12	30	0	0.7496	0.0017	0
78	M	2	27	0.5	11.5468	0.0277	1
40	F	8	30	0.5	0.6167	0.0015	0
53	F	16	30	0	1.8463	0.0041	1
65	M	13	25	0.5	6.1852	0.0151	1
63	M	11	23	0.5	6.7760	0.0139	1
63	M	16	27	0.5	6.6873	0.0163	1
74	M	6	13	1	57.0252	0.1186	3
72	F	6	27	0.5	3.2569	0.0082	1
70	F	14	20	1	15.8820	0.0446	2
75	F	6	20	0.5	6.5138	0.0169	1
70	F	6	26	0.5	4.1284	0.0102	1
31	M	16	30	0.5	0.7865	0.0019	0
54	F	12	29	0.5	1.2629	0.0031	1
76	F	12	20	1	20.5938	0.0518	2
52	F	12	11	2	4.5419	0.0103	0
80	F	6	29	0.5	17.5584	0.0448	2
78	F	0	23	0.5	36.4019	0.0871	2
76	F	7	22	0.5	9.7411	0.0276	1
73	F	13	23	0.5	3.2384	0.0081	1
61	F	6	28	0	2.9061	0.0060	1
68	F	8	0	2	34.1826	0.0854	3
71	F	12	29	0.5	3.1461	0.0071	1
55	M	6	22	1	3.7960	0.0087	1
47	F	12	29	0	0.7976	0.0021	1
76	M	16	25	0.5	15.6457	0.0348	2
60	F	12	29	0	2.3854	0.0051	1
58	F	6	24	0.5	2.2156	0.0053	1
54	F	12	26	0	3.7406	0.0078	1
78	M	16	29	0	10.3024	0.0215	2
77	M	4	26	0.5	5.9008	0.0143	1
58	M	12	29	0.5	21.4652	0.0459	2
78	F	6	21	0.5	27.8335	0.0813	2
70	F	16	30	0	8.7700	0.0183	2
63	F	16	29	0.5	5.9599	0.0143	1
47	M	18	30	0	0.9859	0.0021	0
61	F	6	28	0.5	0.7297	0.0017	0
76	F	19	28	0.5	32.1377	0.0838	3
75	M	6	28	0.5	23.8765	0.0562	2
63	M	8	30	0	3.9807	0.0080	1
68	F	12	29	0.5	6.8063	0.0181	2

61	F	23	27	0	3.3344	0.0081	1
41	F	16	28	0.5	0.7459	0.0017	0
63	M	9	29	0.5	4.8964	0.0137	1
75	M	18	29	0.5	1.2370	0.0030	0
66	F	6	14	1	12.4922	0.0342	2
79	F	1	14	1	2.5664	0.0062	0
61	F	9	30	0	2.5959	0.0062	0
67	F	12	27	0.5	2.7473	0.0067	0
67	M	12	29	0	17.0895	0.0405	3
57	F	16	30	0	0.7651	0.0017	0
64	F	7	30	0.5	3.2089	0.0073	1
48	F	16	27	0.5	1.2998	0.0029	0
70	M	11	23	1	6.5197	0.0174	1
73	M	6	30	0.5	7.9724	0.0178	1
71	M	6	30	0.5	3.7480	0.0081	1
69	M	9	27	0.5	3.8846	0.0088	1
53	F	16	26	0.5	2.0457	0.0043	1
66	F	6	29	0	5.7937	0.0142	1
61	F	17	23	0.5	10.0809	0.0242	2
79	F	12	24	0.5	10.0410	0.0286	1
65	M	8	21	1	7.5256	0.0192	1
72	F	6	28	0.5	11.9604	0.0316	1
62	F	9	30	0	4.6490	0.0115	1
82	F	1	10	2	24.8322	0.0678	2
56	F	9	22	0.5	1.3626	0.0034	0
59	M	16	22	1	5.6571	0.0127	0
90	F	6	14	2	25.3647	0.0693	2
74	F	0	18	0.5	15.5349	0.0383	2
78	M	12	20	2	25.4614	0.0674	2
65	M	18	30	0.5	8.7072	0.0202	1
69	F	9	30	0.5	10.9191	0.0259	1
81	F	16	24	0.5	19.9476	0.0443	2
72	F	2	27	0	12.5143	0.0309	3
67	M	6	12	2	4.5530	0.0105	1
66	M	16	28	0.5	5.3063	0.0109	1
83	F	0	17	0.5	5.0811	0.0125	1
77	M	16	23	0.5	3.7236	0.0092	1
65	M	14	30	0.5	5.8233	0.0153	1
71	M	12	25	0.5	7.5883	0.0158	1
76	M	16	26	0.5	16.6833	0.0429	2
76	F	6	25	0.5	9.8076	0.0249	2
80	F	0	16	0.5	25.3647	0.0732	3
52	M	16	25	0.5	2.5664	0.0056	0

59	M	7	12	2	4.3149	0.0113	1
74	F	18	30	0.5	71.2335	0.1978	1
75	M	6	28	0.5	7.4702	0.0170	1
76	M	16	29	0.5	3.9696	0.0075	1
78	F	0	15	0.5	5.0220	0.0142	1
65	F	9	23	1	52.2248	0.1592	3
67	F	12	29	0.5	17.5503	0.0438	2
85	F	4	25	0.5	2.7436	0.0083	1
67	F	6	21	0.5	4.3868	0.0112	1
78	M	16	26	0.5	11.2551	0.0237	1
69	F	9	24	0.5	24.8403	0.0663	2
76	M	16	26	0.5	30.6894	0.0737	2
74	F	2	16	1	22.5767	0.0664	2
84	F	9	28	0.5	9.1134	0.0276	1
82	F	0.5	13	1	5.8639	0.0149	1
75	F	0	13	1	9.9516	0.0236	1
64	F	23	29	0.5	2.6709	0.0064	0
50	F	14	29	0.5	1.1078	0.0031	0
75	F	6	25	0.5	8.4340	0.0206	1
70	F	2	25	0	3.7813	0.0090	1
64	F	16	30	0.5	2.0605	0.0048	1
76	M	12	30	0.5	21.8825	0.0496	2
75	M	12	29	0.5	12.5254	0.0248	2
76	F	7	19	1	26.1955	0.0618	2
74	M	7	19	1	1.6063	0.0045	0
62	F	16	17	0.5	5.0811	0.0117	1
63	M	6	27	0.5	16.9824	0.0441	1
70	M	16	30	0.5	2.1343	0.0051	1
76	F	9	27	0.5	2.7289	0.0070	0
79	M	6	16	1	16.8605	0.0348	2
67	M	12	16	0.5	28.1046	0.0715	3
79	M	6	25	0.5	8.0241	0.0183	1
84	M	9	25	0.5	18.8804	0.0439	2
71	M	6	19	1	40.9032	0.0990	3
69	M	12	18	1	13.5556	0.0282	1
59	M	12	30	0.5	16.6582	0.0363	2
79	F	8	21	0.5	16.1552	0.0378	2
71	F	0.5	25	0.5	11.8976	0.0322	2
71	F	6	21	0.5	29.2936	0.0909	3
61	F	9	30	0	1.0266	0.0027	1
79	F	16	20	1	13.3858	0.0315	2
58	F	0.5	27	0.5	3.6040	0.0085	0
40	F	16	29	0.5	1.0524	0.0025	0

56	F	12	29	0.5	1.9165	0.0049	1
72	F	9	19	1	22.2850	0.0669	3
77	F	3	29	0.5	5.8344	0.0147	1
71	F	12	26	0.5	28.4864	0.0717	2
82	F	12	2	3	41.4054	0.1188	2
68	F	16	27	0.5	31.8415	0.0796	2
75	F	0.5	28	0	15.5903	0.0374	2
64	M	25	29	0.5	5.8048	0.0123	1
67	F	16	27	0.5	2.5073	0.0057	1
55	F	16	27	0.5	3.3160	0.0074	1
75	M	12	27	0.5	7.0935	0.0175	1
72	F	0	17	0.5	18.7068	0.0420	2
51	F	14	29	0	1.5878	0.0034	0
66	F	9	29	0.5	5.7753	0.0153	1
64	F	6	24	0.5	5.0183	0.0121	1
79	F	6	24	0.5	29.8438	0.0709	3
61	M	12	27	0.5	11.4951	0.0253	1
50	F	12	29	0	2.5435	0.0060	1
76	M	16	30	0.5	8.5337	0.0187	1
55	F	18	30	0	1.4364	0.0033	1
75	M	16	18	1	33.8872	0.0638	3
62	F	14	29	0.5	2.0531	0.0043	1
78	F	2	17	1	5.8011	0.0163	2
75	M	8	29	0.5	5.1844	0.0139	1
61	F	16	26	0.5	2.5073	0.0057	0
62	F	8	26	0.5	12.0897	0.0251	2
78	F	12	28	0.5	24.6741	0.0693	2
61	M	14	25	0	3.1904	0.0061	1
70	F	13	30	0.5	5.2317	0.0125	1
73	F	9	20	0.5	16.3510	0.0400	2
73	M	6	26	0.5	17.1190	0.0393	2
56	F	12	30	0	1.5140	0.0033	0
60	M	14	29	0.5	7.8173	0.0170	1
74	M	12	30	0.5	8.7663	0.0203	1
77	F	1	22	0.5	43.1565	0.1107	1
59	F	12	25	0.5	2.2710	0.0055	1
77	F	9	18	0.5	5.8491	0.0155	1
78	F	12	26	0.5	37.4691	0.0759	2
58	M	18	27	0.5	2.3596	0.0053	1
81	M	0.5	20	0.5	50.9642	0.1065	3
57	M	16	29	0.5	2.0679	0.0040	0
71	M	12	28	0.5	4.1653	0.0093	0
76	M	16	28	0.5	12.4589	0.0255	1

73	F	16	14	1	5.5833	0.0148	1
78	F	12	24	0.5	9.8445	0.0247	1
82	F	6	20	0.5	7.2339	0.0188	2
60	F	12	30	0	2.2931	0.0055	0
57	F	12	28	0.5	1.9172	0.0040	1
27	F	16	28	0	1.5177	0.0035	0
78	F	6	24	0.5	19.5488	0.0533	3
60	F	9	25	0.5	1.9829	0.0047	0
77	M	17	30	0.5	23.6476	0.0593	2
83	F	12	27	0.5	44.0826	0.1454	3
81	M	0.5	21	0.5	15.4908	0.0324	2
62	M	12	26	0.5	4.2391	0.0109	1
77	F	1	17	1	23.8027	0.0627	2
49	F	14	29	0	1.6765	0.0033	1
71	F	7	28	0.5	4.8152	0.0116	1
76	F	12	25	0.5	10.5129	0.0250	2
62	M	18	29	0.5	4.6084	0.0102	1
74	M	0.5	26	0.5	46.0914	0.1014	3
81	F	6	13	1	3.8883	0.0115	1
63	F	9	24	0.5	6.3809	0.0153	1
81	M	18	26	0.5	6.1630	0.0170	1
49	M	12	28	0.5	2.9356	0.0055	1
61	F	8	29	0.5	6.9828	0.0160	1
67	F	6	27	0.5	5.8454	0.0130	1
75	M	12	29	1	2.5885	0.0066	0
71	F	14	27	0.5	3.1830	0.0076	0
75	F	12	23	0.5	18.9875	0.0530	2
73	M	16	24	0.5	26.1032	0.0509	3
84	M	16	28	0.5	17.0009	0.0473	2
52	F	10	25	0.5	2.3965	0.0057	0
70	F	4	24	0.5	8.2161	0.0187	1
61	M	18	30	0.5	4.8410	0.0106	1
81	F	0	14	1	7.2708	0.0208	1
61	F	16	28	0.5	1.3995	0.0032	1
67	M	14	23	0.5	9.6784	0.0210	0
73	F	2	26	0.5	2.8839	0.0077	1
55	F	6	23	0.5	1.5398	0.0039	1
69	F	0	17	1	9.3830	0.0242	1
78	F	9	25	0.5	17.6877	0.0492	2
69	F	9	20	0.5	7.0308	0.0154	2
61	M	18	29	0	1.9165	0.0041	0
77	F	12	30	0.5	12.2263	0.0302	1
73	F	1	12	0.5	25.1246	0.0692	3

68	F	16	25	0.5	3.0169	0.0081	1
68	M	12	25	0.5	20.9062	0.0535	2
74	M	16	25	0.5	26.1475	0.0567	2
38	M	16	28	0.5	1.6100	0.0031	0
73	F	14	30	0.5	3.5966	0.0100	1
64	M	16	26	0.5	6.8166	0.0132	1
81	M	16	19	1	29.2234	0.0659	2
68	F	14	21	1	3.9917	0.0085	1
73	M	7	25	0.5	5.0663	0.0141	1
75	M	18	22	0.5	14.2905	0.0300	2
71	M	1	29	0.5	28.7249	0.0561	3
68	F	9	26	0.5	1.0709	0.0032	1
70	M	12	20	3	6.8277	0.0148	1
70	M	7	16	1	6.8018	0.0164	1
73	M	16	9	1	47.6903	0.0994	3
64	F	6	19	0.5	21.0295	0.0547	2
71	M	6	30	0.5	11.3844	0.0216	1
78	F	8	26	0.5	16.0297	0.0481	3
82	F	0.5	26	0	35.3015	0.0786	3
79	F	12	18	1	19.4195	0.0516	2
72	F	6	25	0.5	4.9149	0.0125	1
54	F	18	27	0.5	2.8212	0.0057	1
82	F	12	29	0.5	28.7619	0.0740	3
63	F	12	30	0.5	1.9940	0.0042	1
78	F	16	23	0.5	10.3571	0.0262	2
72	M	13	21	0.5	5.3580	0.0119	0
87	F	12	24	0.5	7.6733	0.0170	1
72	M	1	24	0.5	38.9757	0.0946	3
70	F	9	27	0.5	1.9940	0.0053	1
74	M	16	23	0.5	8.9325	0.0217	1
43	F	14	30	0.5	6.0559	0.0130	1
53	M	12	20	1	12.5992	0.0230	1
78	M	5	30	0.5	5.5205	0.0108	1
73	F	3	30	0	6.0854	0.0144	1
55	F	12	25	0.5	0.6410	0.0014	0
69	M	6	21	0.5	4.3056	0.0107	1
80	F	6	27	0.5	11.5579	0.0297	1
84	F	12	30	0.5	19.3863	0.0493	2
76	F	6	27	0.5	17.7468	0.0448	2
78	F	6	21	0.5	15.0659	0.0402	2
62	F	6	21	0.5	16.9824	0.0432	2
74	M	18	20	1	17.0562	0.0444	2
71	F	6	23	0.5	1.3766	0.0035	1

70	M	16	21	0.5	10.4723	0.0213	1
79	F	9	24	0.5	2.9283	0.0083	1
80	F	0	12	0.5	17.2874	0.0530	2
58	F	11	29	0.5	3.2904	0.0080	0
49	M	16	25	0.5	1.4290	0.0027	0
68	F	6	29	0.5	9.6230	0.0234	2
62	F	18	30	0.5	1.0967	0.0026	0
55	F	14	17	1	7.1120	0.0167	1
82	F	0	18	0.5	5.9636	0.0169	1
73	M	16	25	0.5	19.0392	0.0439	2
63	F	9	30	0.5	1.9866	0.0048	0
82	M	6	28	0.5	16.3805	0.0409	2
48	F	6	29	0.5	2.2303	0.0051	1
51	F	16	30	0.5	2.6624	0.0064	1
67	F	1	15	1	4.7635	0.0114	1
79	F	12	26	0.5	15.1664	0.0343	2
75	F	0.5	26	0.5	9.7744	0.0221	1
60	F	9	25	0.5	1.8345	0.0043	1
79	M	12	20	0.5	30.3497	0.0715	3
75	F	12	28	0.5	34.4411	0.0804	3
46	F	14	30	0.5	3.5043	0.0068	1
39	F	18	29	0.5	1.0856	0.0026	0
87	M	16	23	0.5	6.6984	0.0164	1
60	F	6	29	0.5	3.7591	0.0074	1
70	F	12	27	0.5	1.8648	0.0047	1
44	M	10	24	0.5	2.3005	0.0055	0
61	F	6	22	0.5	14.0209	0.0318	2
80	F	6	28	0.5	11.5764	0.0314	2
71	M	12	29	0.5	10.1843	0.0246	1
73	M	12	24	0.5	28.3889	0.0606	3
83	F	0.5	21	0.5	4.6490	0.0128	1
72	F	9	24	0.5	7.6031	0.0200	1
75	F	0	18	0.5	11.5875	0.0322	1
77	M	9	25	1	14.7853	0.0342	2
56	F	7	24	0.5	6.1187	0.0138	1
82	F	2	17	0.5	6.0781	0.0174	1
62	M	16	28	0.5	1.9497	0.0043	1
77	M	18	27	0.5	11.9309	0.0280	1
76	M	18	20	1	31.1953	0.0829	3
64	M	12	29	0.5	6.7501	0.0132	1
82	M	12	29	0.5	4.1690	0.0093	1
70	M	16	16	1	14.2949	0.0379	2
73	F	6	21	1	22.2961	0.0500	2

66	F	11	28	0.5	3.0043	0.0075	1
71	M	16	29	0	6.0079	0.0141	2
56	F	18	30	0	1.8943	0.0040	0
69	M	16	23	1	9.5898	0.0192	1
76	F	6	25	1	7.1253	0.0178	1
80	F	6	24	0.5	8.0056	0.0204	1
63	F	2	10	1	4.0582	0.0116	1
54	M	14	23	0.5	1.1743	0.0031	1
64	M	9	30	0.5	16.4839	0.0314	1
65	F	6	27	0.5	10.5018	0.0248	1
75	M	12	17	1	25.9961	0.0737	3
57	M	13	24	0.5	7.4702	0.0157	1
75	M	16	28	1	31.2839	0.0598	2
76	M	16	28	0.5	4.0397	0.0095	1
83	M	16	19	1	22.9386	0.0509	2
61	M	9	28	0.5	2.7030	0.0056	0
65	M	16	30	0	5.9414	0.0128	2
62	M	9	30	0.5	4.8558	0.0112	1
78	M	8	30	0.5	13.3969	0.0274	1
55	F	12	5	2	5.2325	0.0135	1
60	M	16	30	0	1.9497	0.0039	0
82	F	4	13	1	21.3995	0.0526	2
66	F	3	26	0.5	8.8291	0.0201	1
65	F	6	29	0	2.3190	0.0054	1
56	M	18	30	0.5	2.3522	0.0051	0
66	M	9	29	0.5	11.0262	0.0221	1
26	M	16	29	0.5	0.6979	0.0014	0
74	F	0	16	0.5	18.8767	0.0478	2
49	F	18	30	0.5	2.8913	0.0058	0
77	F	16	27	0.5	12.3666	0.0328	2
81	F	12	27	0.5	6.0079	0.0150	1
52	M	16	30	0.5	2.1306	0.0037	0
77	F	9	17	0.5	11.7499	0.0320	1
48	M	16	29	0.5	2.7695	0.0054	0
58	F	6	23	0.5	3.5080	0.0084	1
49	F	11	27	0	2.3190	0.0054	0
75	M	16	29	0.5	11.7160	0.0259	1
59	F	12	30	0.5	2.1454	0.0040	1
64	F	6	27	0.5	62.5217	0.1487	1
58	F	12	29	0.5	1.3995	0.0035	0
79	M	6	26	0.5	23.2119	0.0610	2
57	F	12	29	0	6.2442	0.0155	1
72	F	16	27	0.5	17.8022	0.0462	3

77	F	6	25	1	20.2097	0.0530	2
72	F	24	30	0.5	12.2817	0.0324	0
82	F	6	28	0.5	2.9873	0.0080	0
62	M	16	29	0	3.2754	0.0064	0
73	F	0.5	22	0.5	3.1092	0.0076	1
73	F	2	19	0.5	14.3658	0.0384	2
64	F	14	23	0.5	13.8104	0.0317	2
46	F	12	19	1	11.2145	0.0311	2
56	F	12	30	0.5	1.7835	0.0045	0
64	F	8	25	0.5	2.5139	0.0060	1
64	F	6	19	0.5	13.5741	0.0354	2
89	M	9	22	0.5	7.7397	0.0201	1
73	F	14	23	1	4.3241	0.0114	1
78	F	1	25	0.5	9.0986	0.0249	2
80	F	12	29	0.5	8.9509	0.0231	1
90	F	1	14	1	41.9076	0.1086	3
73	F	9	29	0	26.0441	0.0646	2
50	M	16	30	0.5	2.3116	0.0041	1
81	M	18	21	0.5	84.9046	0.2155	2
56	F	12	27	0.5	1.5472	0.0040	1
78	F	0.5	23	0.5	14.8370	0.0361	2
80	F	0.5	9	2	7.6696	0.0221	1
72	M	6	24	0.5	13.0682	0.0314	2
82	M	25	4	R	16.6353	0.0424	2
62	F	6	29	0	1.3663	0.0036	0
56	F	16	30	0.5	1.0561	0.0024	0
75	F	6	28	0.5	12.1672	0.0276	2
72	F	14	30	0	2.1085	0.0050	1
65	F	12	29	0.5	8.0905	0.0188	2
65	F	6	30	0	2.5294	0.0062	1
69	F	16	29	0.5	4.9850	0.0139	1
74	F	4	27	0.5	22.9201	0.0581	2
74	M	16	27	0.5	7.5071	0.0194	1
83	F	0.5	16	1	13.6701	0.0348	3
67	F	12	26	0.5	7.8653	0.0154	1
72	F	9	27	0.5	45.3750	0.1039	3
77	M	12	23	0.5	37.2734	0.1064	3
77	F	6	23	0.5	5.7937	0.0146	1
73	M	18	23	0.5	3.4046	0.0089	0
69	F	12	30	0	4.5973	0.0112	1
59	F	8	29	0.5	2.8618	0.0077	1
55	F	16	18	0.5	5.2731	0.0137	1
74	F	6	25	0.5	9.2463	0.0236	1

59	F	8	27	0	5.5796	0.0124	1
67	F	17	23	0.5	5.9821	0.0158	2
59	F	0	25	0.5	4.8706	0.0105	1
68	F	12	30	0.5	1.9829	0.0044	1
66	F	12	27	0	4.2761	0.0111	1
59	M	12	16	1	32.0446	0.0741	3
72	M	12	30	0	10.7566	0.0218	1
58	M	16	27	0.5	4.4644	0.0104	0
77	F	0.5	17	1	11.4545	0.0297	2
81	F	0.5	19	0	6.1888	0.0144	1
62	F	NA	30	0	1.2020	0.0025	0
58	M	NA	30	0	1.6750	0.0035	0
53	F	NA	30	0	1.5300	0.0036	0
34	M	NA	30	0	1.6660	0.0035	0
37	F	NA	30	0	0.8330	0.0020	0
52	F	NA	30	0	0.8480	0.0020	0
34	F	NA	30	0	0.4640	0.0011	0
56	F	NA	30	0	0.5940	0.0015	0
60	F	NA	30	0	1.8970	0.0056	0
53	F	NA	30	0	1.0220	0.0022	0
49	F	NA	30	0	1.7810	0.0037	0
67	F	NA	30	0	1.0750	0.0029	0
36	F	NA	30	0	0.7890	0.0027	0
70	F	NA	30	0	1.4120	0.0035	0
50	F	NA	30	0	1.0040	0.0026	0
64	M	NA	30	0	1.9620	0.0048	0
63	M	NA	30	0	1.3230	0.0030	0
62	M	NA	30	0	1.4710	0.0035	0
