# Supplementary figures and images for: Diagnostic performance of deep learning-based automatic white matter hyperintensity segmentation for classification of the Fazekas scale and differentiation of subcortical vascular dementia
Source: PLoS One. 2022 Sep 15;17(9):e0274562. doi: 10.1371/journal.pone.0274562 (PMC9477348; doi:10.1371/journal.pone.0274562)

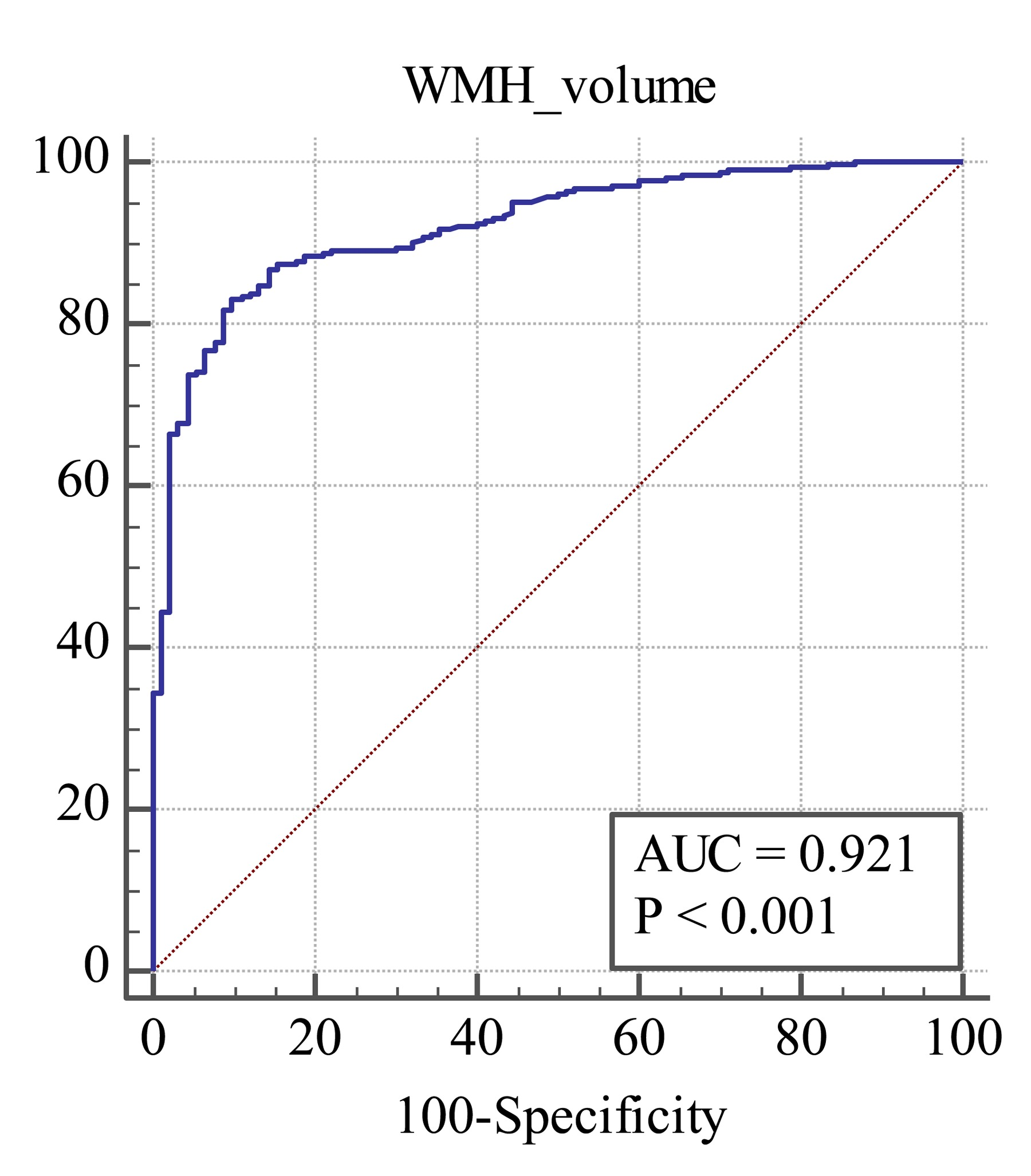

Supplement: S1 Fig — (TIF) [file pone.0274562.s002.tif]

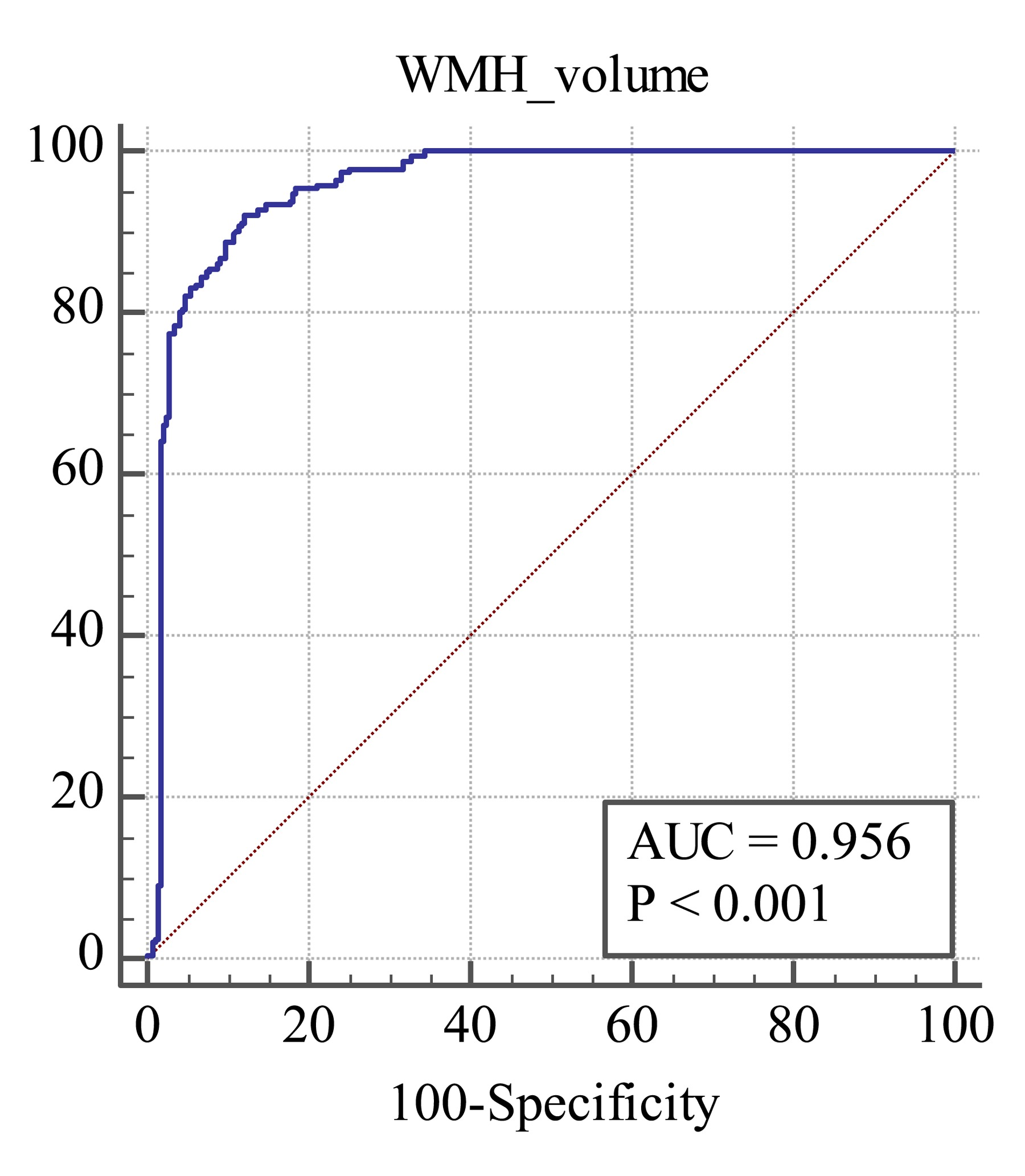

Supplement: S2 Fig — (TIF) [file pone.0274562.s003.tif]

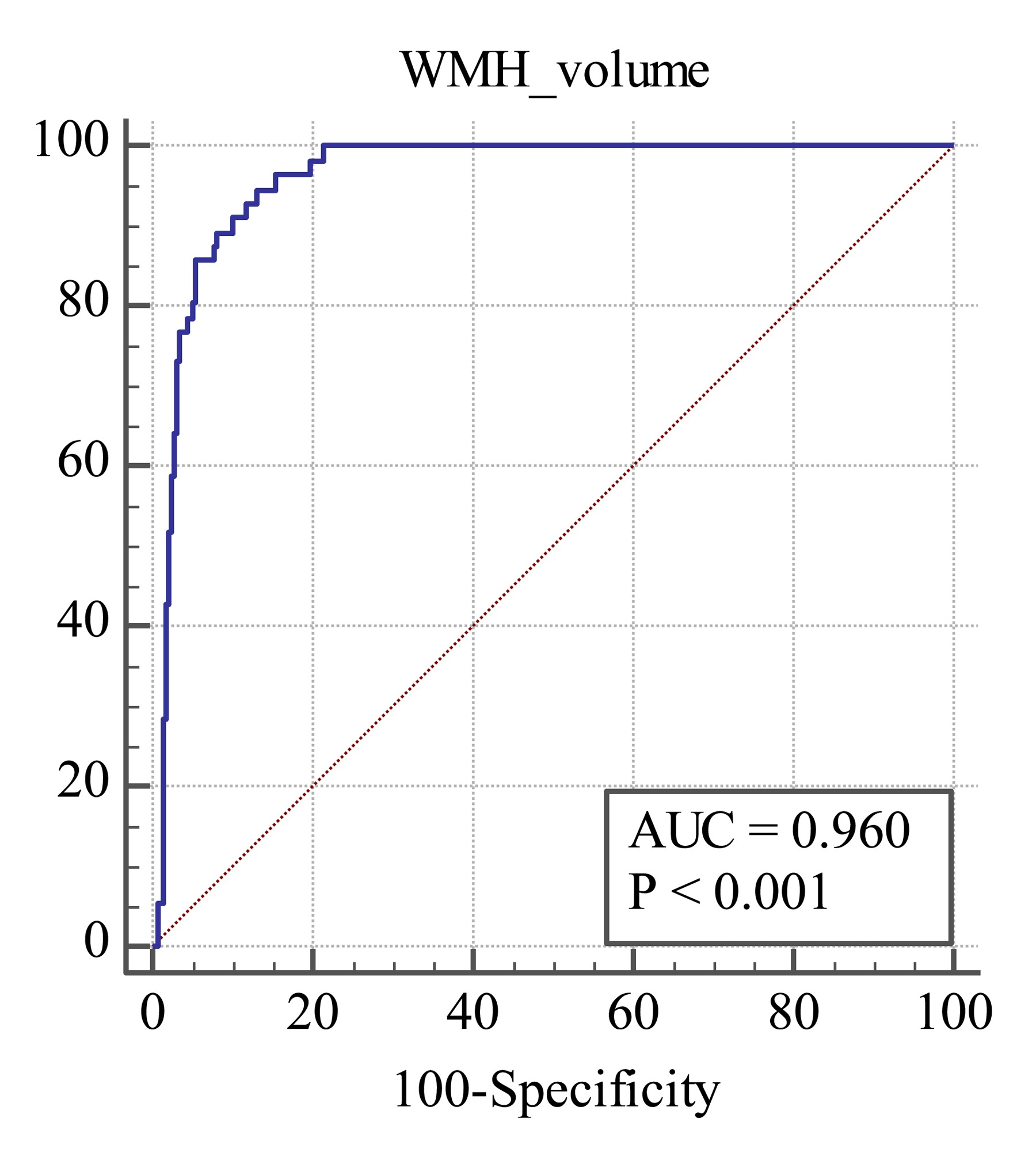

Supplement: S3 Fig — (TIF) [file pone.0274562.s004.tif]

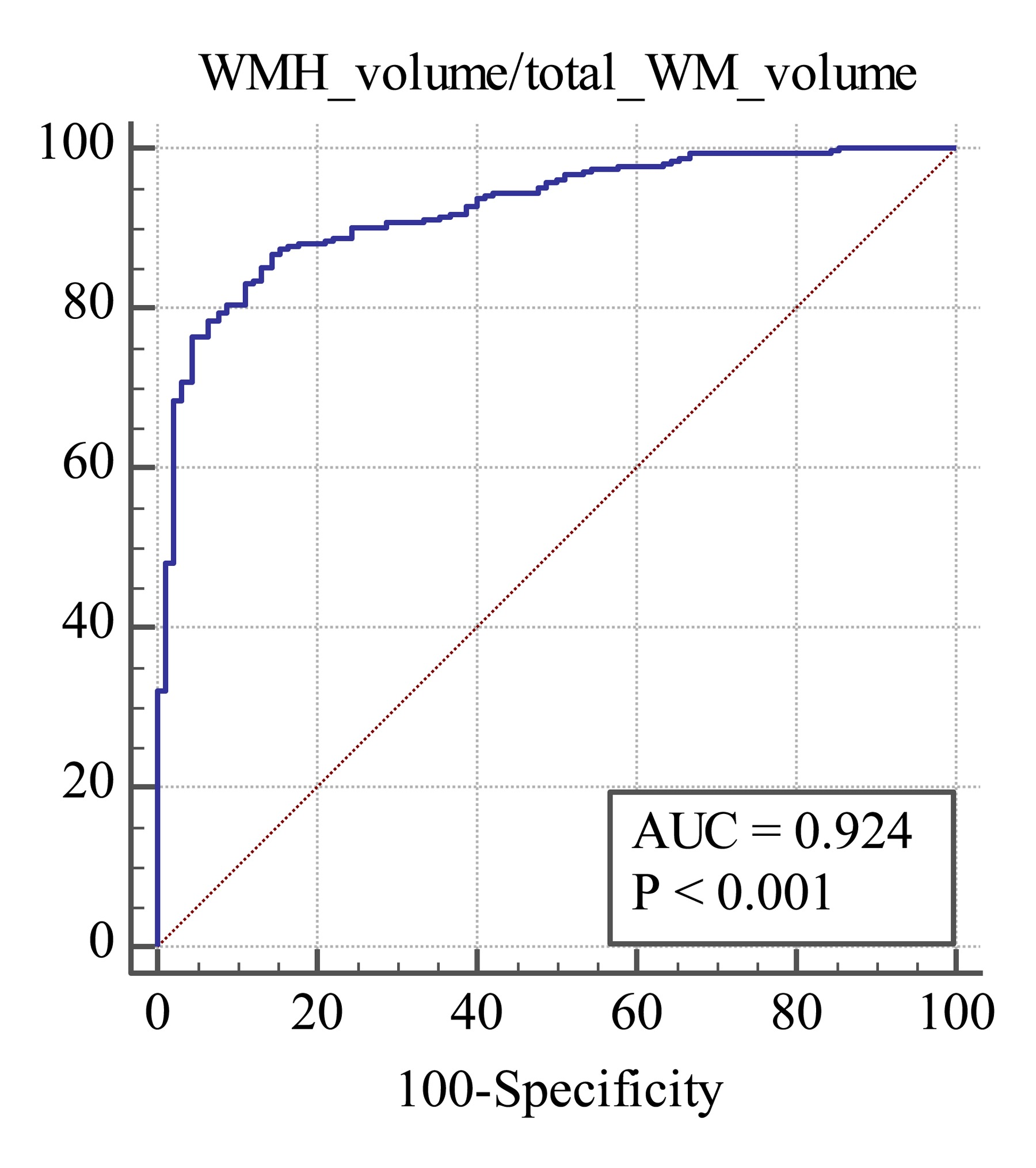

Supplement: S4 Fig — (TIF) [file pone.0274562.s005.tif]

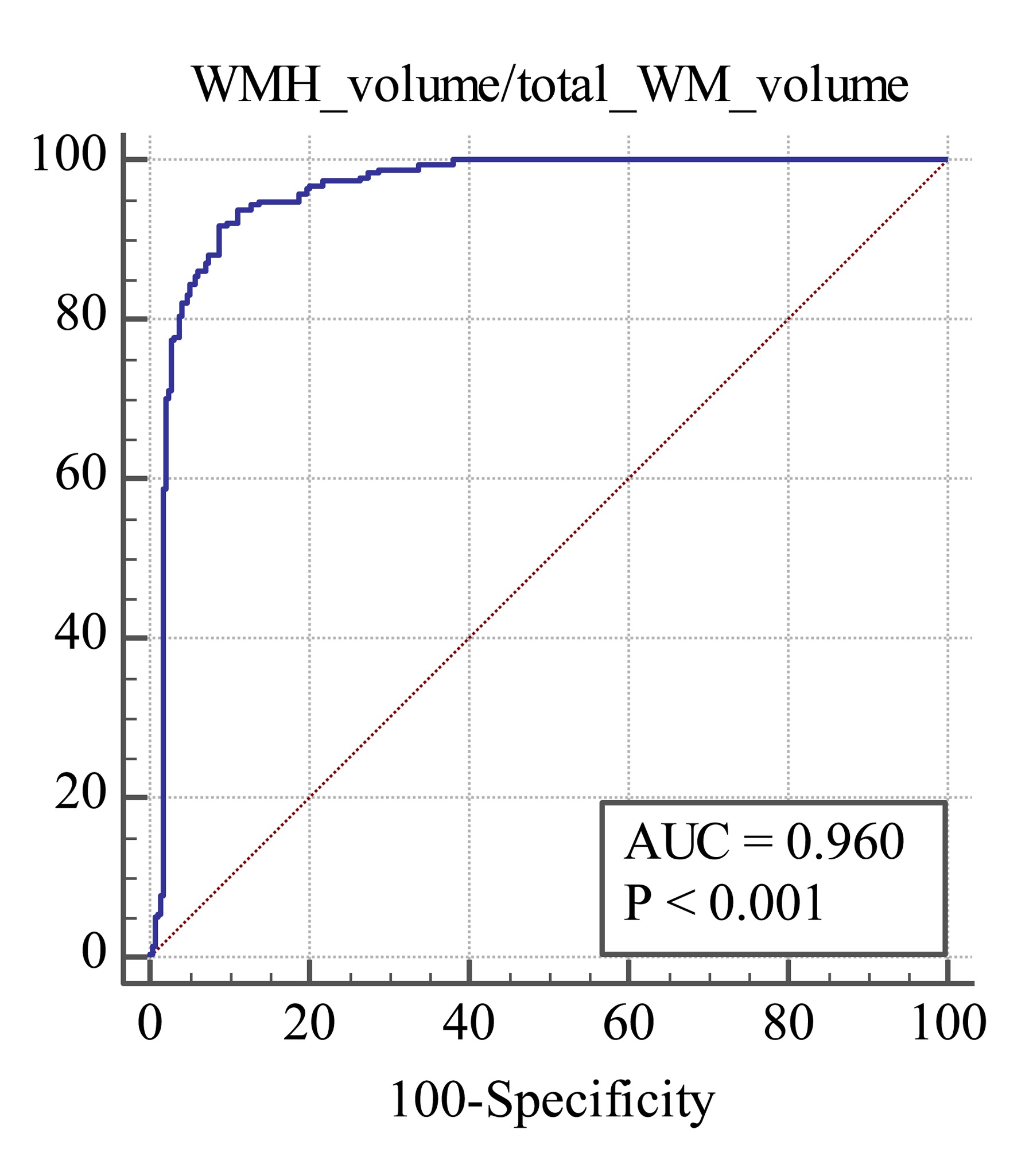

Supplement: S5 Fig — (TIF) [file pone.0274562.s006.tif]

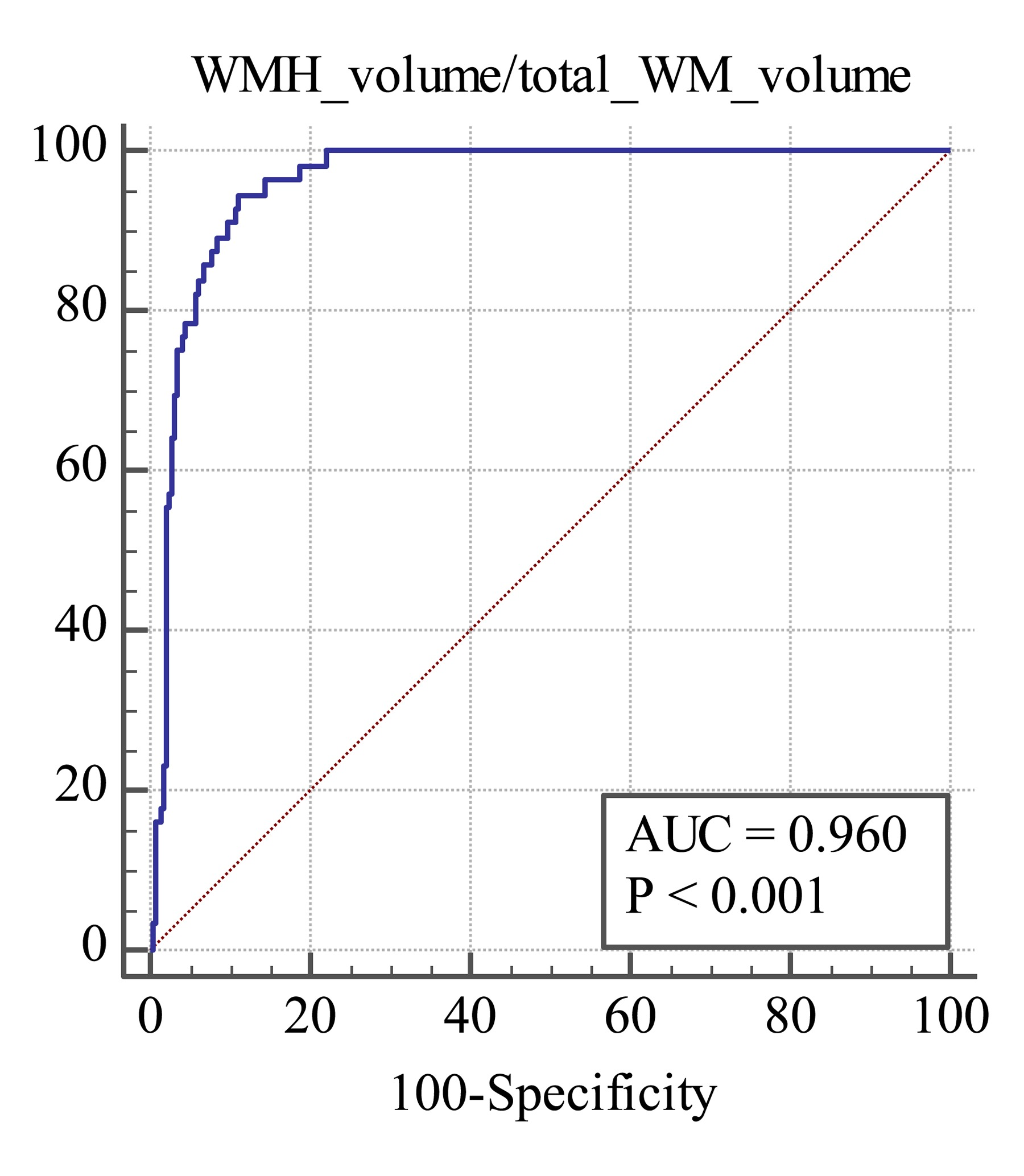

Supplement: S6 Fig — (TIF) [file pone.0274562.s007.tif]
